# Architecture of epigenetic reprogramming following Twist1-mediated epithelial-mesenchymal transition

**DOI:** 10.1186/gb-2013-14-12-r144

**Published:** 2013-12-24

**Authors:** Gabriel G Malouf, Joseph H Taube, Yue Lu, Tapasree Roysarkar, Shoghag Panjarian, Marcos RH Estecio, Jaroslav Jelinek, Jumpei Yamazaki, Noel J-M Raynal, Hai Long, Tomomitsu Tahara, Agata Tinnirello, Priyanka Ramachandran, Xiu-Ying Zhang, Shoudan Liang, Sendurai A Mani, Jean-Pierre J Issa

**Affiliations:** 1 Department of Leukemia, the University of Texas MD Anderson Cancer Center, Houston, TX, USA; 2 Department of Medical Oncology, Groupe Hospitalier Pitié - Salpêtrière, Assistance Publique Hopitaux de Paris, Faculty of Medicine Pierre et Marie Curie, Institut Universitaire de Cancérologie, Paris, France; 3 Department of Translational Molecular Pathology, The University of Texas MD Anderson Cancer Center, Houston, TX, USA; 4 Department of Bioinformatics, the University of Texas MD Anderson Cancer Center, Houston, TX, USA; 5 Metastasis Research Center, The University of Texas MD Anderson Cancer Center, Houston, TX, USA; 6 Fels Institute for Cancer Research and Molecular Biology, Temple University, Philadelphia, PA, USA

## Abstract

**Background:**

Epithelial-mesenchymal transition (EMT) is known to impart metastasis and stemness characteristics in breast cancer. To characterize the epigenetic reprogramming following Twist1-induced EMT, we characterized the epigenetic and transcriptome landscapes using whole-genome transcriptome analysis by RNA-seq, DNA methylation by digital restriction enzyme analysis of methylation (DREAM) and histone modifications by CHIP-seq of H3K4me3 and H3K27me3 in immortalized human mammary epithelial cells relative to cells induced to undergo EMT by Twist1.

**Results:**

EMT is accompanied by focal hypermethylation and widespread global DNA hypomethylation, predominantly within transcriptionally repressed gene bodies. At the chromatin level, the number of gene promoters marked by H3K4me3 increases by more than one fifth; H3K27me3 undergoes dynamic genomic redistribution characterized by loss at half of gene promoters and overall reduction of peak size by almost half. This is paralleled by increased phosphorylation of EZH2 at serine 21. Among genes with highly altered mRNA expression, 23.1% switch between H3K4me3 and H3K27me3 marks, and those point to the master EMT targets and regulators *CDH1, PDGFRα* and *ESRP1*. Strikingly, Twist1 increases the number of bivalent genes by more than two fold. Inhibition of the H3K27 methyltransferases EZH2 and EZH1, which form part of the Polycomb repressive complex 2 (PRC2), blocks EMT and stemness properties.

**Conclusions:**

Our findings demonstrate that the EMT program requires epigenetic remodeling by the Polycomb and Trithorax complexes leading to increased cellular plasticity. This suggests that inhibiting epigenetic remodeling and thus decrease plasticity will prevent EMT, and the associated breast cancer metastasis.

## Background

Epithelial-mesenchymal transition (EMT) is known to promote cellular plasticity during the formation of the mesoderm from epiblasts and the neural crest cells from the neural tube in the developing embryo as well as during adult wound healing [1]. During EMT, epithelial cells lose their epithelial characteristics and acquire mesenchymal morphology, which facilitates cellular dissociation and migration. Similar to embryo development, neoplastic cells have been shown to reactivate EMT leading to cancer metastasis [2]. Induction of EMT is also involved in the development of resistance to cytotoxic chemotherapy and targeted agents [3-5]. In addition, EMT imparts stem cell properties to differentiated cells [6]. Since cancer cells seem to acquire stem cell properties dynamically in response to the tumor microenvironment and become differentiated at distant sites, it has been suggested that major epigenetic remodeling would occur during EMT to facilitate metastasis. Although DNA methylation changes at specific loci have been established during EMT [7,8], changes in the global DNA methylation landscape are not well understood. Indeed, a recent report demonstrated that DNA methylation is largely unchanged during EMT mediated by transforming growth factor beta (TGF-β) [9], while another showed that EMT is associated with specific alterations of gene-related CpG-rich regions [10]. Moreover, another report showed a striking difference in DNA methylation in non-small cell lung cancers between mesenchymal-like tumors and epithelial-like tumors, which display a better prognosis and exhibit greater sensitivity to inhibitors of epidermal growth factor receptor [11].

In addition to DNA methylation, EMT mediates epigenetic reprogramming through widespread changes in post-translational modifications of histones [12]. However, it is unknown if switches in histone marks coordinate EMT and, in particular, whether genome regulation by Polycomb group (PcG) and Trithorax group (TrxG) proteins are critical regulators for this transition, as is the case for germ cell development and stem cell differentiation. Indeed, the TrxG complex activates gene transcription by inducing trimethylation of lysine 4 of histone H3 (H3K4me3) at specific sites, whereas the PcG complex represses gene transcription by trimethylation of lysine 27 on histone H3 (H3K27me3). Of note, a subset of promoters in embryonic stem cells are known to have methylation at both H3K4 and H3K27 (the bivalent state), which poise them for either activation or repression in different cell types upon differentiation [13]. However, the transcriptional dynamics and the role of those bivalent genes in differentiated cells and during EMT are still poorly understood.

The development of genome-wide sequencing expanded our understanding of the plasticity of DNA methylation during differentiation of embryonic stem cells, tumorigenesis and metastasis [14,15]. During the differentiation of embryonic stem cells into fibroblasts, the majority of DNA methylation changes occur outside of core promoters in partially methylated domains (PMDs), which represent large hypomethylated regions covering approximately 40% of our genome [14]. Using genome-wide DNA methylation analyses, these PMDs have been shown to be hypomethylated in adipose tissue [16], placenta [17,18], cultured breast cancer cells [19] and neuronal cells [20], as well as in several cancer types [15]. PMDs overlap with domains of H3K27me3 and/or H3K9me3, transcriptional-repression associated histone marks, in IMR90 fibroblasts [14]. In breast cancer, widespread DNA hypomethylation occurs primarily at PMDs in normal breast cells [21]. However, whether DNA methylome changes during EMT recapitulate tumor formation remains unknown.

EMT is often a transient process, with changes in gene expression, increased invasiveness, and acquisition of stem cell properties such as increased tumor initiation, metastasis and chemotherapeutic-resistance. It’s transient nature suggests that significant features of an EMT could be regulated by epigenetic fluidity triggered by key transcription factors and signaling events in response to an alteration in the tumor microenvironment. We present genome-wide changes in DNA methylation and histone modifications in H3K4me3 and H3K27me3 following the induction of EMT by the ectopic expression of the transcription factor Twist1 using immortalized human mammary epithelial cells (HMLE) [2]. Additionally, we compared the Twist1-expressing HMLE cells, hereafter HMLE Twist, cultured in a monolayer to the same cells cultured as mammospheres (MS), which enriches for cells with stem cell properties [22]. We found that EMT is characterized by major epigenetic reprogramming required for phenotypic plasticity, with predominant alterations to polycomb targets. Moreover, we have shown that inhibition of the H3K27 methyltransferases EZH2 and EZH1 - part of the polycomb repressive complex 2 (PRC2) - either by short hairpin RNA (shRNA) or pharmacologically, blocks EMT and stemness properties.

## Results

### Aberrant promoter DNA methylation induced by epithelial-mesenchymal transition is cell-type specific and regionally coordinated

**Figure 1:**
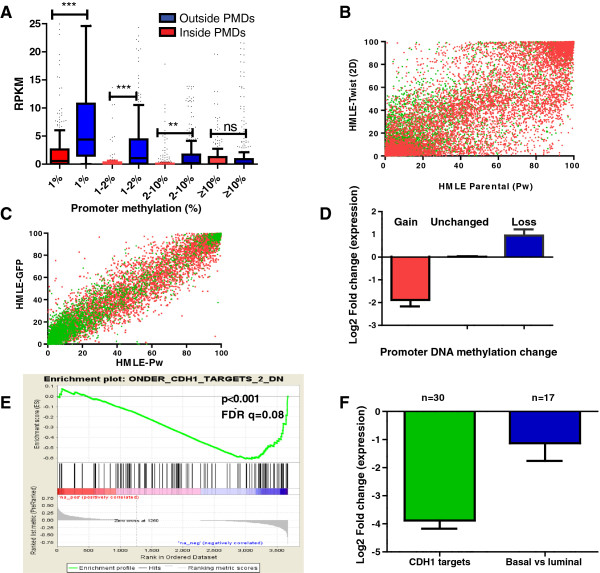
**DNA Methylation changes occurring following epithelial-mesenchymal transition. (A)** Box-plot for gene expression levels according to promoter DNA methylation level of genes located in partially methylated domains (PMDs) (red) and outside PMDs (blue) in human mammary epithelial cells (HMLE). The x-axis represents promoter % methylation and y-axis represents normalized expression level. *** *P* <0.0001, ** *P* <0.001, ns: non-significant. **(B)** Correlation of DNA methylation level of CpG sites in HMLE vector cells (Pw) (x-axis) and HMLE Twist cells (y-axis), showing dramatic changes in DNA methylation following EMT. **(C)** Correlation of DNA methylation level of CpG sites in HMLE vector cells transduced with two different ‘control’ vectors Pw (x-axis) and GFP (y-axis) showing no change in DNA methylation. **(D)** Box plots of average gene expression levels of genes with a gain, loss or no change in DNA methylation. Note that a gain of DNA methylation (increase to ≥2% DNA methylation from gene promoters which were fully unmethylated, ≤1%) was associated with 3.8-fold decrease of their expression levels, while demethylation of gene promoters leading to fully unmethylated promoters (≤1%) was associated with an increase in their gene expression levels by about 2-fold. **(E)** GSEA showing that genes losing gene body methylation following EMT are enriched for genes which are down-regulated in a *CDH1*-knockdown model of EMT (*P* <0.0001). The bottom graph represents the rank-ordered, non-redundant list of genes. Genes on the far right (blue) correlated most strongly with decreased gene expression in the *CDH1*-knockdown model of EMT. FDR, false discovery rate. **(F)** Box plots showing decreased expression levels of genes losing gene body methylation following Twist-induced EMT in two different models: knockdown of *CDH1* and basal breast cancers compared to luminal breast cancers. y-axis represents the log2-fold change of gene expression.

According to the EMT model of cancer progression, epithelial cells undergo a phenotypic change during the sequential progression of primary tumors towards metastasis, accompanied or not by DNA methylation changes [9,10]. Although aberrant promoter methylation on some specific promoters was previously reported [23], genomic distribution and genome-wide mapping of methylome changes during this process remains unclear. To identify DNA methylation changes in EMT, we used digital restriction enzyme analysis of methylation (DREAM), which yields highly quantitative genome-wide DNA methylation information [24]. Because small changes in DNA methylation could be important, we focused the analysis on sites with a threshold of 100-fold coverage per sample (average = 1,178 tags per CpG site). By examining approximately 30,000 CpG sites spanning the promoters of around 5,000 genes (Table S1 in Additional file 1), we observed the expected relationships: lower DNA methylation at CpG islands (CGI) compared to non-CGI; lowest methylation around the transcription start site (TSS) (Figure S1A in Additional file 2); and a strong negative-correlation between promoter DNA methylation and gene expression (Spearman’s R < −0.50, *P* <0.0001), independent of Twist1 expression. Interestingly, the quantitative nature of the data allowed us to establish that genes with completely unmethylated promoters (methylation ≤1%) were highly expressed in comparison to promoters with an appreciable level of methylation (>1%; Figure 1A). As there is an important overlap between PMD regions in different tissues [20,25], we analyzed gene expression according to the localization of genes in PMDs. As expected, genes located within PMDs had lower baseline expression in our model, regardless of methylation (Figure 1A), and average gene body methylation was lower for CpG sites located within PMDs compared to those located outside PMDs (21.5% versus 40% respectively, *P* <0.0001; Figure S1B in Additional file 2). Thus, we conclude that even low levels of DNA methylation at promoters are inhibitory for gene expression and genes within PMDs tend towards lower expression.

Expression of Twist1 caused a dramatic change in DNA methylation both at CGIs and at non-CGIs (Figure 1B) whereas no changes were seen between cells with independent control vectors, suggesting that the methylation changes observed are related to Twist1 expression and not to random clonal drift (Figure 1C). To study the impact of these changes on gene expression, we focused on completely unmethylated genes (<1%) and identified 90 genes out of 3,008 (3%) that switched from <1% to >2% with an average gain of 5.4% DNA methylation. As expected, this was associated with about a four-fold decrease in the expression of these genes (*P* <0.0001; Figure 1D). The gain of methylation was higher in genes located within PMDs (12%; 37 out of 309) versus outside PMDs (2%; 53 out of 2,699; χ2 test, *P* <0.0001). Conversely, there were 39 genes that become unmethylated upon Twist1 expression, concomitant with around a two-fold increased expression of the respective genes (Figure 1D), such as *FOXC2*, a master regulator of EMT [26-28]. In contrast with promoter methylation, promoter hypomethylation was more frequent outside PMDs (4.6%; 31 out of 670) than within PMDs (1.8%; 8 out of 455; *P* <0.02). Gene ontology (GO) analysis for genes with methylation change associated with gene expression change showed enrichment for cell adhesion genes such as *DSCAM*, *NID1* and *NID2* (*P* = 0.002), consistent with the functional change of motility and migration of mesenchymal cells. Moreover, we found an enrichment of genes (*P* = 5e^-05^) involved in calcium binding protein coding genes (that is, *FBN1*, *NPNT*), suggesting a functional role for orchestrated calcium-binding proteins in EMT that may represent a novel therapeutic target for controlling cell plasticity. Collectively, these data suggest that induction of EMT by Twist1 results in a moderate change in the DNA methylation of core promoters.

### Twist1 promotes global demethylation outside of core promoters

To understand the global methylation and demethylation changes that occur in response to induction of EMT by Twist1, we focused on 4,903 CpG sites with a threshold detection of a minimum of 100 tags that had a baseline methylation ≥70%, as is typical of most of the genome [14]. Among these 4,903 CpG sites, one fifth (18.6%) lost DNA methylation following EMT (Table S2 in Additional file 1). We obtained comparable results using thresholds of 10 tags, and three tags per CpG site, covering 7,081 and 11,117 CpG sites respectively (data not shown). This widespread hypomethylation was mainly observed in PMDs (*P* <0.0001; Table S2 in Additional file 1) and was independent of the genomic CpG location in repeats and lamina-associated domains (Figure S2A-C in Additional file 3). Moreover, we found decreased methylation of repetitive elements at short interspersed nuclear elements, long interspersed nuclear elements and satellite repeats (Figure S2D in Additional file 3). Concomitant with global PMD demethylation, we also observed focal hypermethylation specific to those promoters (Figure S1C,D in Additional file 2), consistent with data recently reported in colon cancer [25]. These data suggest that methylome change during EMT is reminiscent of methylome changes observed in cancer.

To understand the functional relevance of gene body methylation changes following the induction of EMT by Twist1, we performed Gene Set Enrichment Analysis (GSEA). GSEA is a computational method that assesses whether a defined set of genes (herein, gene bodies) shows statistically significant difference between two conditions (herein, between epithelial and mesenchymal states) [29]. While there was no enrichment for any pathway associated with gain of gene body methylation, GSEA reveals enrichment for gene body hypomethylation for EMT targets in the *CDH1*-knockdown model (*P* <0.0001 [30]; Figure 1E), and for MIR34B and MIR34C targets [31] (Table S3 in Additional file 1). Concomitantly, average expression level of those hypomethylated genes was lower after knockdown of *CDH1*, as well as in basal-like compared to luminal-like breast cancer subtypes [32,33] (*P* <0.004; Figure 1F). Collectively, these data suggest that following the induction of EMT by Twist expression, Twist reprograms the genome by demethylating gene bodies of epithelial cell-specific genes, leading to a decrease of their expression levels.

### Twist1 increases the number of promoters with H3K4me3 by more than one fifth

**Figure 2:**
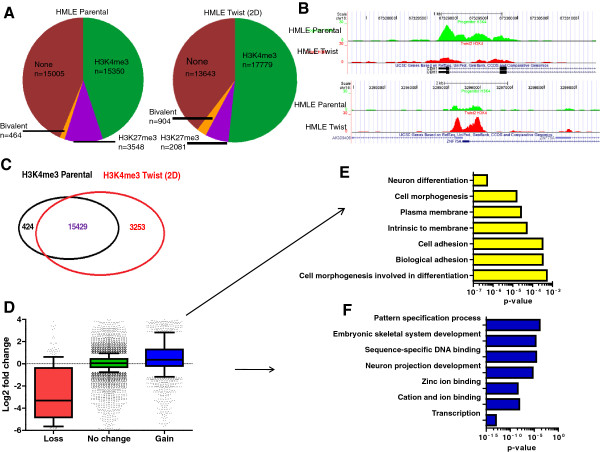
**H3K4me3 dynamic modifications are coupled with transcriptional changes related to epithelial-mesenchymal transition genes. (A)** Pie chart showing the distribution of H3K4me3 and H3K27me3 marks in human mammary epithelial cells (HMLE) vector cells and HMLE Twist cells. **(B)** Landscape of H3K4me3 for *CDH1* (loss of H3K4me3 in vector cells) and *ZNF75A* (gain of H3K4me3) in HMLE Twist cells. **(C)** Venn diagram of H3K4me3 at gene promoters in HMLE vector cells and HMLE Twist cells. **(D)** Box plots for gene expression changes in genes losing or gaining the H3K4me3 mark. **(E)** Gene ontology analysis using DAVID for genes losing the H3K4me3 mark. The x-axis represents the *P*-value levels and y-axis the gene ontology pathways. **(F)** Gene ontology analysis using DAVID for genes gaining the H3K4me3 mark. The x-axis represents the *P*-value levels and y-axis the gene ontology pathways.

Overall, the number of genes marked by H3K4me3 and also by both H3K4me3 and H3K27me3 (bivalent) was increased following Twist1-induced EMT (Figure 2A,B). Specifically, we observed that more than 20% (3,253 out of 15,853) of tallied genes acquired H3K4me3 but less than 3% (424 out of 15,853) of genes lost H3K4me3 (Figure 2C). As expected, acquisition of H3K4me3 was associated with increased mRNA expression whereas loss of H3K4me3 led to reduced expression of the corresponding genes (Figure 2D). GO analysis indicated that the set of genes that lose H3K4me3 is significantly enriched for genes associated with cell adhesion and differentiation (Figure 2E). Conversely, gain of H3K4me3, which is mediated by the TrxG complex, was found in EMT-promoting transcription factors, including zinc-ion binding proteins (i.e. *ZNF75A)*, highlighting the dramatic effect of TrxG machinery in chromatin remodeling during EMT (Figure 2F). GSEA showed enrichment for estrogen receptor (ESR1) targets (*P* <0.0001, false discovery rate (FDR) *q* <0.05) within genes losing H3K4me3 (Table S4 in Additional file 1). As a result, ESR1 targets in HMLE vector cells lose the active mark H3K4me3 consistent with the three-fold decrease of ESR1 expression in HMLE Twist cells (data not shown). Importantly, genes losing H3K4me3 were also enriched for genes down-regulated in blood vessel cells from the wound site, suggesting epigenetic conservation of the EMT process between wound healing and cancer (Table S4 in Additional file 1). Thus, EMT is accompanied by a widespread gain in H3K4me3-mediated gene activation, and loss of H3K4me3 at ESR1 targets.

### Switches between H3K4me3 and H3K27me3 modulate transcriptional dynamics

**Figure 3:**
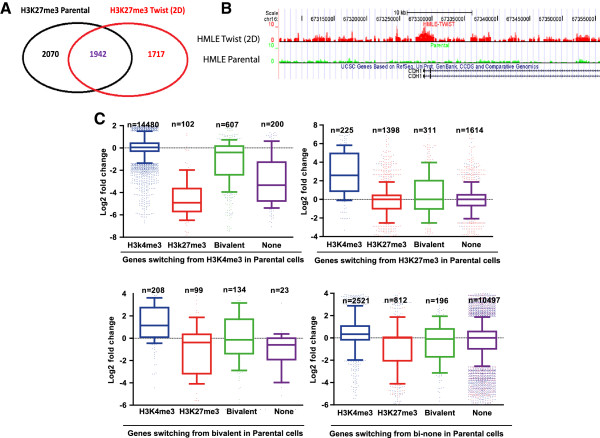
**H3K27me3 switches orchestrate a mesenchymal cell-type specific gene expression signature. (A)** Venn diagram of genes marked by H3K27me3 in human mammary epithelial cells (HMLE) vector cells and HMLE Twist cells. **(B)** Landscape of H3K27me3 mark in *CDH1* gene (gain of H3K27me3 mark in HMLE Twist cells). **(C)** Box-plot of gene expression (the bars represent 10% and 90% extremes) for genes switching in HMLE vector cells from H3K4me3, H3K27me3, bivalent or neither marks to other histone mark combinations in HMLE Twist cells.

Using chromatin immunoprecipitation sequencing (ChIP-seq) for the H3K27me3 repressive histone modification, we found that the genomic distribution of H3K27me3 was significantly reduced in HMLE Twist cells (250 Megabases in vector cells compared to 153 and 138 Megabases in HMLE Twist cells cultured in monolayer and spheres, respectively; data not shown). This is consistent with the notion that cells that have undergone EMT are less differentiated and have acquired stem cell properties [6]. Given these changes in the landscape of H3K27me3, we investigated switches between H3K27me3 and H3K4me3 during EMT. Expression of Twist1 caused a loss of H3K27me3 in more than 50% of the genes marked by H3K27me3 in HMLE cells (Figure 3A,B). Of the 2,070 genes that lost H3K27me3, approximately 11% (225 out of 2,070) switched to H3K4me3 (Figure 3C) and we found that transcription of these genes was dramatically induced (around five-fold; *P* <0.0001). Conversely, the genes that lost H3K27me3 without gain of H3K4me3 had no average change in their respective gene expression (Figure 3C). Overall, 102 genes switched from H3K4me3 to H3K27me3 after Twist1-induced EMT, and were transcriptionally repressed by more than 32 fold (Figure 3C). These chromatin switches were associated with differential gene expression, particularly at typical EMT markers. For example, the repression of E-cadherin expression during EMT correlated with a switch from H3K4me3 to H3K27me3 (Figure 3B). Conversely, gain of N-cadherin expression correlated with a switch from H3K27me3 to H3K4me3. Strikingly, the same interplay between H3K4me3 and H3K27me3 occurs for master genes involved in the EMT process, such as *PDGFRα*, which is essential for Twist1 to promote tumor metastasis via invadopodia [34], and the splicing regulator *ESRP1*, which is repressed by Snail1 to promote EMT [35] (Table S5 in Additional file 1). Among genes with highly altered expression during EMT increasing or decreasing at least nine fold), 23.1% of them switched between H3K4me3 and H3K27me3 marks, as compared to only 2.8% for genes without highly altered expression (Figure S3 in Additional file 4). Altogether, these data suggest that an epigenetic program orchestrated by TrxG or PcG complexes regulate key EMT genes.

**Figure 4:**
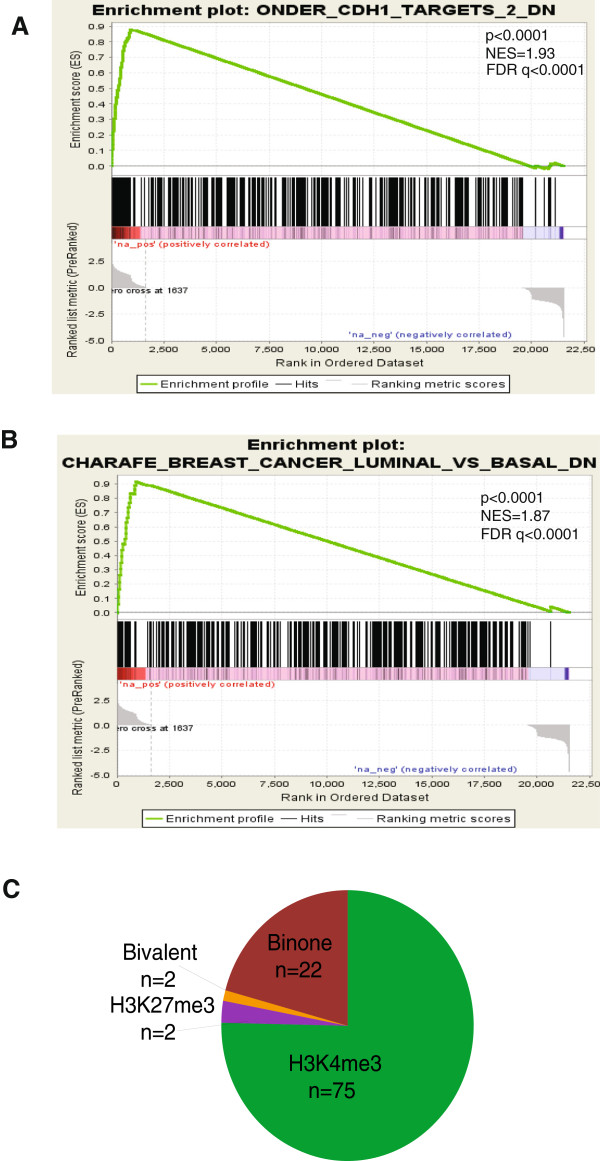
**Gene set enrichment analysis of H3K27me3 marks. (A)** Gene set enrichment analysis showing positive enrichment for H3K27me3 marks in genes which are down-regulated by the *CDH1*-knockdown model of EMT (*P* <0.0001). The bottom graph represents the rank-ordered, non-redundant list of genes. Genes on the far left (red) correlated the most with decreased gene expression following *CDH1*-knockdown. The vertical black lines show the position of each of the genes of the studied gene set in the ordered, non-redundant data set. The green curve is related to the enrichment score curve. **(B)** Gene set enrichment analysis showing positive enrichment for H3K27me3 marks in genes distinguishing luminal from basal like breast cancer (*P* <0.0001). **(C)** Pie chart showing that the majority of downregulated genes in the *CDH1*-knockdown model of EMT which gain H3K27me3 in Twist1-cells were also pre-marked by H3K4me3 in vector cells. FDR, false discovery rate; NES, normalized enrichment score.

During EMT, parallel to the dramatic loss of H3K27me3 occupancy in nearly 50% of genes (n = 2,070) marked in HMLE vector cells, mesenchymal cells gained H3K27me3 at 1717 genes. GSEA analysis showed that these genes were enriched for functional categories in a cell-type specific manner. Indeed, the set of genes which gained H3K27me3 is related to genes down-regulated in a previously described *CDH1* knockdown model of EMT [30] (Figure 4A) and to genes with low expression in basal-like as compared to luminal-like breast cancer cell lines [32] (*P* <0.0001; Figure 4B; Table S6 in Additional file 1). Of interest, the majority of genes down-regulated by *CDH1* knockdown and which gained H3K27me3 in Twist1-induced cells were pre-marked by H3K4me3 in HMLE cells, highlighting the importance of TrxG and PcG switches in defining cell identity during EMT (Figure 4C). Furthermore, GSEA revealed that genes belonging to pathways that gained H3K27me3 were associated with DNA repair and mRNA splicing (Table S6 in Additional file 1). Conversely, genes belonging to pathways that lost H3K27me3 were associated with mitotic pre-metaphase and undifferentiated cancer signature (Table S6 in Additional file 1).

Importantly, we sought to investigate if the changes we observed in Twist cells could be replicated in other EMT model systems such as Snail and TGF-β1-induced model systems. If we found similar findings across multiple EMT models, this would rule out adaptation and suggest that the effect we observed in Twist cells was due to EMT and not necessarily adaptation. In fact, we found that the majority of sites (14 out of 17) demonstrated the same directional change in H3K4me3 and/or H3K27me3 by ChIP-qPCR in HMLE Snail, TGF-β1 and Twist cells as we observed by ChIP-seq in HMLE Twist cells (Figure S4 in Additional file 5). The Pearson correlation coefficients for Snail versus Twist (r = 0.8982, *P* <0.0001), for Snail versus TGF-β1 (r = 0.4613, *P* = 0.006) and for TGF-β1 versus Twist (r = 0.1791, *P* = 0.3108) point to close similarities between Snail- and Twist-induced EMTs in their effects on H3K4me3 and H3K27me3 whereas expression of TGF-β1 has a less similar effect (Figure S5 in Additional file 6). We also observed similar results for the methylation of DNA elements assessed using bisulfite sequencing in the promoters of seven genes randomly chosen out of genes switching between H3K27me3 and H3K4me3 (Figure S6 in Additional file 7, Figure S7 in Additional file 8 and Figure S8 in Additional file 9). Collectively, our data suggest that our a majority of changes due to Twist expression are not due to adaptation but rather shared with cells undergoing EMT through other means.

### Enrichment in bivalent genes upon Twist1 induction

**Figure 5:**
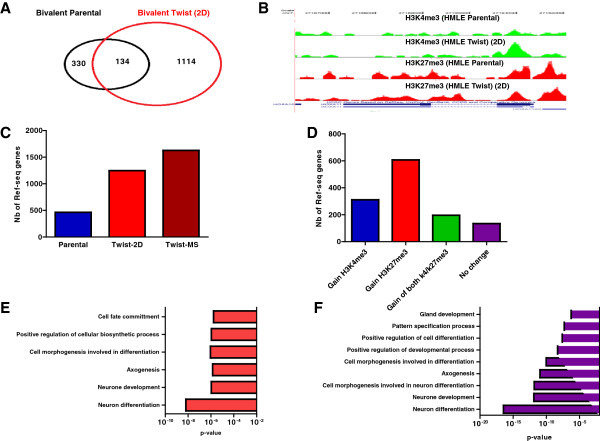
**Bivalent genes are highly enriched in mesenchymal cells. (A)** Venn diagram of bivalent genes in human mammary epithelial (HMLE) vector cells and HMLE Twist- cells. **(B)** Landscape of H3K4me3 and H3K27me3 mark for the homeobox gene *HOXA11*. Note that *HOXA11* is marked by H3K27me3 in both epithelial and mesenchymal cells. In HMLE Twist cells, *HOXA11* gains H3K4me3 mark in addition to H3K27me3 mark. **(C)** Bar graph showing the number of bivalent genes in HMLE vector cells, and HMLE Twist cells in a monolayer (Twist-2D) and sphere culture (Twist-MS). **(D)** Changes in histone modifications among genes becoming bivalent in HMLE Twist cells (Twist-2D) as compared to HMLE vector cells. **(E)** Gene ontology of newly bivalent genes in HMLE Twist cells cultured in monolayer (Twist-2D). **(F)** Gene ontology of newly bivalent genes in HMLE Twist cells cultured in spheres (Twist-MS). 2D, monolayer; MS, mammosphere.

Bivalent genes were characterized initially in stem cells by the co-occurrence of H3K27me3 (repression) and H3K4me3 (activation) at genes which become either transcriptionally active (H3K4me3) or repressed (H3K27me3) upon differentiation [13]. We found that the number of bivalent genes increased by more than 2.7-fold in HMLE Twist cells (n = 1,248; Figure 5A), as was the case for HOX genes (for example, *HOX11*; Figure 5B). The number of bivalent genes was further enriched by almost four fold in stem cell-enriched MS culture (n = 1,628) as compared to baseline of 464 genes in HMLE vector cells (Figure 5C). These data are consistent with the notion that the mesenchymal cells generated via EMT are less differentiated and that this feature is further enriched in MS culture. Overall, of all genes marked by H3K27me3, 34.1% were bivalent (1,249 out of 3,659) in HMLE Twist cells compared to 11.6% in HMLE cells (464 out of 4,012). The majority of bivalent genes in HMLE Twist cells were pre-marked by H3K4me3 or H3K27me3 in HMLE cells (Figure 5D). Thus, Twist-induced chromatin changes indicate a reverse differentiation state, with more ‘poised’ genes and, therefore, greater plasticity. Using GO analysis, we found that the newly bivalent genes in Twist1 cells were enriched for genes involved in neuron differentiation, cell morphogenesis, axonogenesis and cell fate commitment both in monolayer and sphere cultures (Figure 5E,F). This is consistent with the notion that genes involved in differentiation acquire a poised state in less differentiated cells. Of note, more than 50% (825 out of 1,628) of bivalent genes found in MSs were also bivalent in embryonic stem cells.

### Chromatin changes in spheroid cultures

Cells with stem cells properties are known to initiate sphere formation in non-attachment cultures including the cells induced to undergo EMT. Whereas previous work has shown that sphere culture actually decreases the number of bivalent genes [36], we observed an increase in the number of bivalent genes from 464 to 1,628 (Figure 5C). Furthermore, we compared the expression of genes, DNA methylation and histone modifications of HMLE Twist cells cultured in monolayers (two dimensional) or in MS (three dimensional) and found that the DNA methylation in the two states was highly similar (Spearman’s R >0.96, *P* <0.0001; Figure S9A in Additional file 10). By contrast, we found that 2.6% of the genes (849 out of 33,004) increased their expression more than four fold and 2.2% (737 out of 33,004) decreased their expression more than four fold when transitioned from monolayer to MS culture. GSEA analysis revealed positive enrichment for different pathways related to interferon responses (Table S7 in Additional file 1); by contrast, there was negative enrichment (exclusion) for pathways involved in proliferation [37], as well as for genes up-regulated in grade 3 versus grade 1 invasive breast cancer tumors [38] (Table S7 in Additional file 1). Consistent with earlier findings in an ovarian cancer model [36], there was a significant switch toward more genes marked by H3K27me3 in MS cells (3,607) compared to monolayer (2,411; Figure S9B in Additional file 10), but the majority of these genes were already transcriptionally silenced in monolayer culture in response to the overexpression of Twist1. This was also the case for the 186 genes switching from H3K4me3 in monolayer to H3K27me3 in MS. Remarkably, there was a loss of H3K4me3 mark in 2,894 genes when cultured in MS compared to monolayer (Figure S9B in Additional file 10). We then asked whether histone switches between HMLE vector and HMLE Twist cells cultured in spheres were consistent with gene expression changes despite culture condition, and found that this was the case (Figure S9C in Additional file 10). These data suggest that changes in H3K4me3 and H3K27me3 distribution accompany a Twist1-driven EMT, either cultured in monolayer or spheres.

### Chromatin interplay between DNA methylation and histone modifications

**Figure 6:**
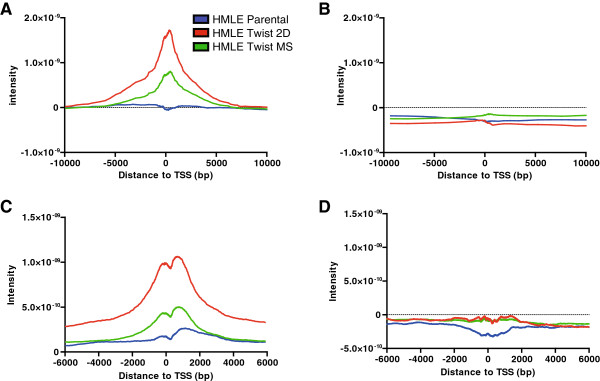
**Correlation between DNA methylation and H3K27me3 mark. (A)** During epithelial-mesenchymal transition (EMT), gain of DNA methylation (≥2%) at selected promoters in human mammary epithelial (HMLE) Twist cells cultured either in monolayer (green) or spheres (red) and which were completely unmethylated (≤1%) in HMLE vector cells (blue) is accompanied by gain of the H3K27me3 mark. The x-axis represents distance from the CpG sites gaining DNA methylation to the transcription start sites (TSS). The y-axis represents average intensity of H3K27me3 peaks. **(B)** During EMT, loss of DNA methylation (≤2%) of selected promoters in HMLE Twist cells cultured either in monolayer (green) or spheres (red) and which were methylated in HMLE vector cells (blue) is not associated with change of H3K27me3 distribution. The x-axis represents distance from the CpG sites losing DNA methylation to the TSSs. The y-axis represents average intensity of H3K27me3 peaks. **(C)** Distribution of H3K27me3 marks in partially methylated domain (PMDs) in HMLE Twist cells cultured in a monolayer (green) and spheres (red) as compared to HMLE vector cells. **(D)** Distribution of H3K27me3 marks outside PMDs in HMLE Twist cells either cultured in a monolayer (green) or as spheres (red) as compared to HMLE vector cells.

Because our data suggest that both DNA methylation and histone modifications are altered throughout the genome following Twist1-induced EMT, we examined the relationship between these different modifications. We found that low-level gain of DNA methylation in promoter regions was associated with a significant increase in H3K27me3 in both Twist1 monolayer and MS culture (Figure 6A). This was validated by bisulfite sequencing in seven selected gene promoters switching from H3K4me3 in HMLE vector cells to H3K27me3 in HMLE Twist cells (Table S8 in Additional file 1). Conversely, loss of DNA methylation was not associated with loss or gain of H3K27me3 (Figure 6B). Furthermore, both in HMLE and HMLE Twist cells, we found a strong positive correlation between low-level DNA methylation at the promoter and the presence of H3K27me3 (Spearman’s R >0.34, *P* <0.0001). Interestingly, the association between gene silencing and enrichment of H3K27me3 around TSSs was much more pronounced in genes located in PMDs (Figure 6C) than outside PMDs (Figure 6D).

To assess whether there is an opposing correlation between H3K4me3 and DNA methylation, we focused on the gene promoters that gain DNA methylation and decrease gene expression by two fold or more. Overall, 22 out of 30 genes lost their H3K4me3 mark, highly confirming the opposing relationship between DNA methylation and H3K4me3 (data not shown). Conversely, out of the 19 gene promoters losing DNA methylation and gaining expression, six genes gained a *de novo* H3K4me3 mark, while the 13 other genes that already had a H3K4me3 mark in HMLE vector cells kept it in HMLE Twist cells.

### Epigenetic plasticity mediated by EZH2 is required for epithelial-mesenchymal transition

**Figure 7:**
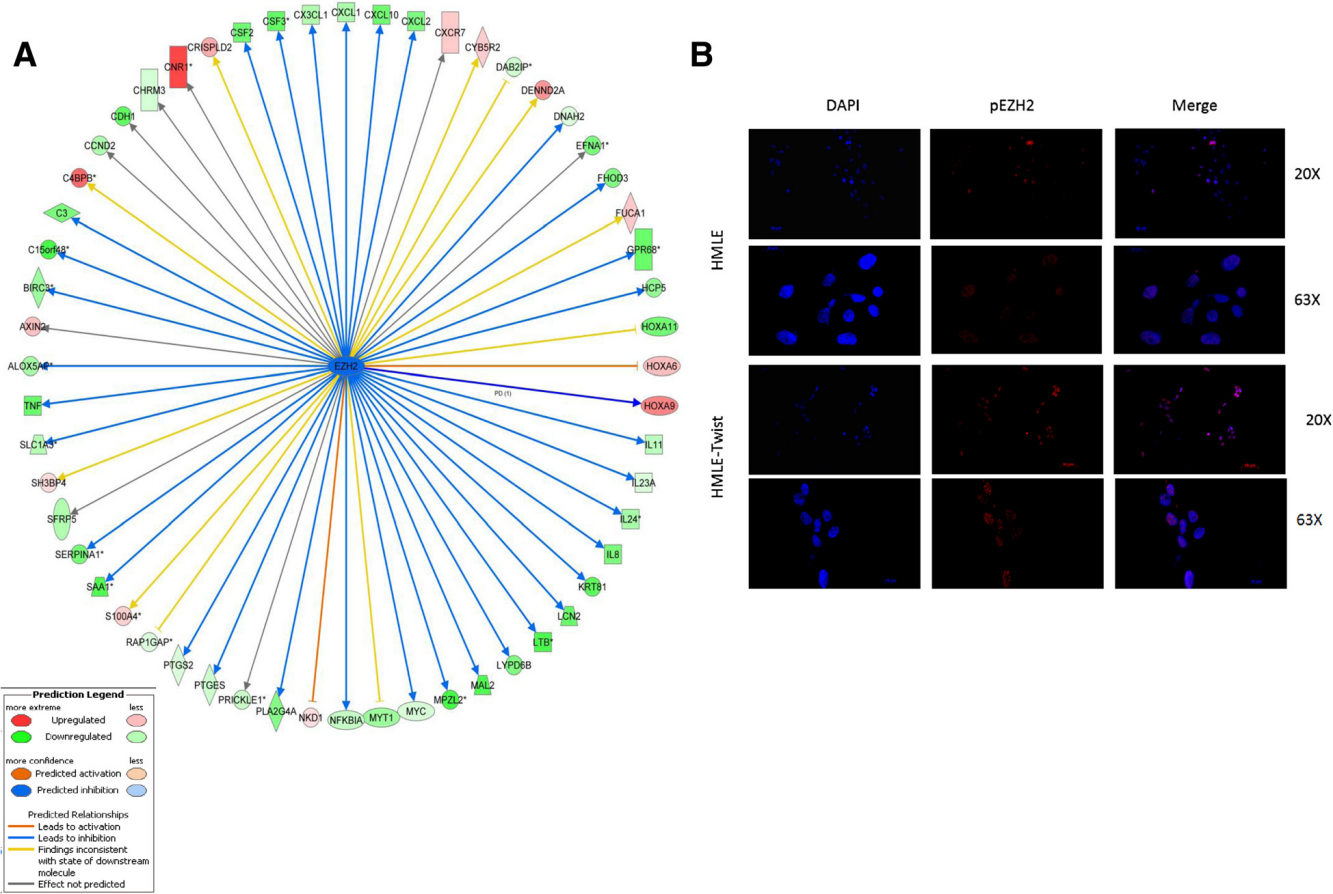
**Phosphorylation of serine residue 21 of EZH2 is increased following epithelial-mesenchymal transition. (A)** Ingenuity Pathway Analysis showing inhibition of EZH2 as a potential upstream regulatory event following Twist-induced epithelial-mesenchymal transition. Overall, 37 target genes were predicted to be inhibited by EZH2. **(B)** Immunofluorescence showing increase of phosphorylation of EZH2 at serine residue 21 in human mammary epithelial (HMLE) Twist cells as compared to HMLE vector cells.

We then asked if there is any chromatin regulator that may explain gene expression changes during EMT. Ingenuity Pathway Analysis was used to investigate chromatin regulators capable of altering major changes in gene expression during EMT. We identified EZH2 as one of the top upstream regulators to be inhibited following EMT (*P* = 8.25e^-11^; Figure 7A; Table S9 in Additional file 1). In our HMLE model, following EMT, we did not observe an alteration in expression of EZH2, which could account for a change in PRC2 activity. Nevertheless, our ChIP-seq revealed a decrease in H3K27me3 peak lengths, presumably mediated by EZH2 by more than half. Furthermore, out of 37 EZH2 target genes identified by IPA as repressed, approximately one third (n = 11) were repressed through gain of H3K27me3; for the remaining 26 genes, this was independent of PRC2 activity. Thus, we reasoned that post-translational modification of EZH2 may account for a decrease in methyltransferase activity and consequent repression of its target genes. In fact, phosphorylation of EZH2 at serine residue 21 is known to significantly affect its function [39,40]. Thus, we used immunofluorescence to examine gain of phosphorylation in EZH2 at serine residue 21 in HMLE Twist cells relative to control cells. We observed a striking increase in phosphorylation of EZH2 in HMLE Twist cells (Figure 7B). This suggests that this post-translational modification may account for decreased trimethylation of H3K27 notwithstanding inhibition of EZH2 target genes through a PRC2-independent mechanism.

**Figure 8:**
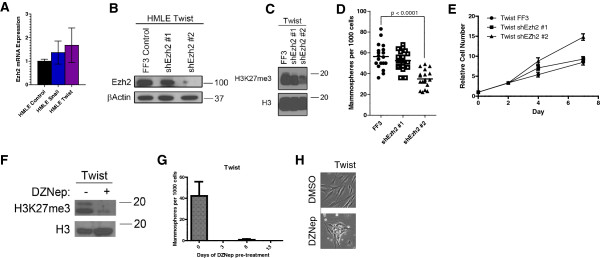
**EZH2 is required for Twist-induced stemness. (A)** Quantitative reverse transcription polymerase chain reaction expression of EZH2 in human mammary epithelial (HMLE) cells expressing the indicated epithelial-mesenchymal transition (EMT) inducers. **(B)** EZH2 protein levels assessed by western blot in HMLE Twist cells transduced by vectors expressing two different short hairpin RNAs (shRNAs) targeting EZH2 and a control vector (FF3). Note that ShRNA #2 was more efficient in reducing EZH2 protein levels as indicated by western blot. **(C)** Histone H3 and H3K27me3 protein levels assessed by western blot indicate that shRNA #2 reduces H3K27me3. **(D)** Mammospheres were counted in HMLE Twist FF3 cells (control) and shEZH2 cells grown in mammosphere-promoting conditions. Spheres greater in size than 50 micrometers after 10 days were counted and a Student’s t-test was performed. **(E)** Count of HMLE Twist FF3 (control) and shEZH2 cells after multiple days in standard cell culture conditions. **(F)** Western blot analysis of H3K27me3 level in HMLE Twist cells before and after treatment with 3-deazaneplanocin A. **(G)** HMLE Twist cells were treated with 2.5 μM of 3-deazaneplanocin A for 8 days followed by 48 hours without drug. Surviving cells were then subjected to a sphere assay in the absence of drug for 8 days. **(H)** The morphology of HMLE Twist cells treated with 2.5 μM 3-deazaneplanocin A is depicted after 8 days of treatment. We observed a change toward an epithelial morphology.

We then asked whether EZH2 is also required for the EMT. We examined the importance of EZH2 in Twist-induced EMT. While the expression of EZH2 was not significantly altered following the expression of Twist1 or other EMT-inducing factors in HMLE cells (Figure 8A), the suppression of EZH2 using shRNA was sufficient to reduce the level of H3K27me3 (Figure 8B,C). In addition, reduction of EZH2 significantly reduced the number of MSs compared to the control shRNA expressing cells (Figure 8D). This reduction in sphere formation was not due to changes in cell growth rates in monolayer culture (Figure 8E) or changes in the expression of EMT-promoting transcription factors (Figure S10 in Additional file 11). Interestingly, knockdown of the EZH2 homolog EZH1 (also a part of the PRC2 complex) mimics the sphere-formation defect similarly to EZH2 knockdown (Additional file 12: Figure S11). Furthermore, reduction of EZH2 protein using 3-deazaneplanocin A [41] was sufficient to reduce both H3K27me3 and sphere formation (Figure 8F,G). Notably, the sphere assay was performed in the absence of the drug, following an 8-day pretreatment and a 48-hour interim period to avoid cytotoxicity during sphere growth. Furthermore, the morphology of HMLE Twist cells following 3-deazaneplanocin A treatment was more epithelial than control-treated cells (Figure 8H). Together, these results indicate that EZH2 plays an essential role in the pathophysiology of EMT through PRC2-dependent and -independent mechanisms.

## Discussion

The findings presented here provide the first comprehensive genome-wide demonstration of the remodeling of the epigenome following Twist1-induced EMT, in terms of DNA methylation as well as trithorax and polycomb-related histone modifications, concurrent with quantitative gene expression analysis by RNA-seq. In particular, we show evidence that genome-wide DNA methylation changes involve focal hypermethylation and global hypomethylation of PMDs, reminiscent of methylome changes observed between normal mammary cells and breast cancers. This highlights, for the first time, that EMT recapitulates DNA methylation changes observed during breast cancer carcinogenesis [21]. As with embryonic stem cell differentiation, the majority of changes occur outside core promoters, suggesting that cell plasticity, even in differentiated cells, involves a profound change in the DNA methylome and histone packaging. These data are in contrast with data reported recently showing unchanged DNA methylation during EMT mediated by TGF-β [9]. One explanation could be that the 36-hour exposure to TGF-β was too short to observe changes in DNA methylation. In addition, our analysis of H3K4me3 and H3K27me3 ChIP data from HMLE Snail, Twist and TGF-β cells indicates that TGF-β cells are more divergent than EMT induced by Snail and Twist, an observation that was initially made using microarray data in Taube *et al*. [33]. Nevertheless, our data provide powerful evidence to support the viewpoint recently brought forward by Pujadas and Feinberg that shifts in distinct regions of the epigenetic landscape, in our case PMDs, undergird cellular plasticity in both developmental and disease contexts [42].

Unexpectedly, our DREAM analysis found that a small gain of promoter DNA methylation is often coupled with a gain of H3K27me3, which strongly suggests that DNA methylation and PcG proteins act together. Indeed, DNA methylation is linked to polycomb protein-mediated repression; however, there is a debate as to whether DNA methylation and PcG proteins act together [43] or independently [44,45] to silence gene expression. Deep sequencing allows more accurate measurement in the low methylation range and the etiology and functional significance of small changes in DNA methylation is unknown. Although DNA methylation could be contributing to gene silencing, it is also possible that silencing by PcG proteins weakens protection against DNA methylation and thus indirectly promotes small increases. It is interesting that remodeling at PMDs was associated with both DNA hypermethylation of promoters and global demethylation; we speculate that a redistribution of TET (ten-eleven-translocation) and DNMT (DNA (Cytosine-5-)-Methyltransferase) proteins may facilitate plasticity during EMT [46].

Most strikingly, cells that had undergone EMT displayed a large increase in the number of bivalent genes. Certainly, these poised genes might help mediate the stem cell properties previously reported in this model. How Twist1 leads to this gain of bivalency deserves further investigation; however, this is the first description of remodeling involving differentiated cells having similarities with the differentiation of embryonic stem cells [13].

Beyond the pure description of the landscape of DNA methylation and histone changes, these data provide a holistic framework for studying EMT-mediated changes in chromatin and gene expression. Indeed, our data show that key EMT markers switch between H3K4me3 and H3K27me3. As an example, we found an opposing histone switch for E-cadherin and N-cadherin, and that the expression change was associated with histones switches but not with DNA methylation changes. In fact, E-cadherin lost H3K4me3 and gained H3K27me3, whereas N-cadherin did the opposite. Moreover, other EMT regulators (different from EMT markers) were also found to be subject to H3K4me3 and H3K27me3 switches, such as *ESRP1* and *PDGF***α**, which are known to be involved in splicing and invadopodia formation respectively [34,47]. Therefore, we speculate that other genes that exhibit histone modification switches are key EMT genes and deserve more focus in the study of this process. Indeed, with the development of deep sequencing and the decreased cost of sequencing, we speculate that alterations in histone landscape may be used in the future as a tool for drug discovery.

Lastly, we have shown that both the epigenetic modifiers EZH2 and EZH1 are essential for the stemness property of cells that have undergone EMT in response to Twist1 expression. EZH2 is a known marker of aggressive breast cancer [48], specifically through an influence on cancer stem cells [49], but a link to EMT has not yet been described. Because the expression of either EZH2 or EZH1 was not significantly altered by EMT, we hypothesize that the EMT-induced changes in the H3K27me3 landscape are mediated primarily by changes in EZH2 or EZH1 localization and function. Of note, the suppression of EZH2 by shRNA and pharmacologically by 3-deazaneplanocin A was sufficient to reduce both H3K27me3 and sphere formation, opening avenues for the use of EZH2 inhibitors to reverse EMT-induced tumor resistance to hypoxia or chemotherapeutics. Further work is called for to detail the mechanisms leading to these changes. Currently, there are active efforts to develop EZH2 inhibitors for cancer therapy, and our data suggest that they may also be useful to suppress epigenetic plasticity and its physiological consequences, such as metastasis and drug resistance.

## Conclusions

We show that induction of EMT results in dramatic alterations in the epigenetic landscape involving significant changes in both DNA methylation (mainly outside core promoters) and histone modifications (that is, an increase in bivalent genes, gene switching between H3K4me3 and H3K27me3) and that these changes contribute to the stem cell properties and increase cellular plasticity. Thus, inhibiting epigenetic remodeling may block plasticity which facilitates EMT and associated breast cancer metastasis.

## Methods

### Characterization of human mammary epithelial cells

HMLE Twist cells were derived as shown in Yang *et al*. [2]. Briefly, we overexpressed Twist1 using retroviral vectors and the transduced cells were selected using puromycin. This method yields a very high transduction rate (>99%). To further confirm the homogeneity of this population of cells, we performed immunofluorescence for *VIM* and *FOXC2* markers, which are known to be induced following EMT (Figure S12 in Additional file 13). Similar results were obtained for HMLE Snail and HMLE TGF-β1 cells ( Figure S13 in Additional file 14).

### Digital restriction enzyme analysis of methylation methods

DREAM was performed as reported previously [50]. Briefly, genomic DNA was sequentially digested with a pair of enzymes recognizing the same restriction site (CCCGGG) containing a CpG dinucleotide, as previously reported. The first enzyme, SmaI, cut only at unmethylated CpG and left blunt ends. The second enzyme, XmaI, was not blocked by methylation and left a short 5′ overhang. The enzymes thus created methylation-specific signatures at the ends of digested DNA fragments. These were deciphered by next-generation sequencing using the Illumina Gene Analyzer II and Hiseq2000 platforms (Illumina, San Diego, CA, USA). Methylation levels for each sequenced restriction site were calculated based on the number of DNA molecules with the methylated or unmethylated signatures. Overall, we acquired around 36 million sequence tags per sample that were mapped to unique CpG sites in the human genome, using version hg18. Details of the DREAM method were previously reported by Challen *et al*. [50].

### Genome annotation of DREAM data and statistical analysis

Genomic regions were defined according to National Center for Biotechnology Information coordinates downloaded from the University of California Santa Cruz website [51] in April 2010. Promoters were defined as regions between −1,000 bp and +1000 bp from TSSs for each RefSeq transcript. Gene bodies were defined as the transcribed regions, +1,000 bp from TSS to the end of the transcription sites for each RefSeq transcript. To calculate promoter methylation, we averaged the methylation level of all CpG sites located between −1000 bp and 1000 bp of the TSS. To estimate the FDR of promoter methylation using our method, we reasoned that a comparison of HMLE cells transduced with Twist1 according to culture conditions (monolayer versus MS) could be used because their methylation was remarkably identical (Spearman’s R >0.96, *P* <0.0001; Figure S3B in Additional file 4). The FDR was 0% for 5% gain of methylation for unmethylated genes (≤1%), and 0.001 (5 out of 4,655) for a difference of 2% of methylation for unmethylated genes (≤1%). Of note, a different transduction of HMLE with a different vector led to a minimal change of promoter methylation with 20 genes out of 3,933 genes gaining 2% methylation at unmethylated loci (≤1%). Using a minimum of 300 tags per SmaI site, only 7 out of 3,933 unmethylated genes gained more than 2% methylation, confirming the method’s high level of precision.

Because the majority of the genome is heavily methylated in contrast to CpG sites related to promoters located in CGI, we used different criteria to analysis DNA methylation changes at the genome level. Arbitrarily, we considered that CpG sites with methylation level ≤10% were unmethylated and a threshold gain of 20% was defined as hypermethylation; conversely, CpG sites with methylation level ≥70% were considered methylated and a threshold loss of 20% was defined as hypomethylation.

For the localization of CpG sites in PMDs or outside PMDs, we downloaded data published for fetal lung fibroblasts (IMR90) [14]. The genes were considered to be located in PMDs if their promoters were located within PMDs. The graphs were prepared using GraphPad Prism 5.0 for Windows, GraphPad Software, San Diego California USA. For the graph of the distribution of CpG sites detected by DREAM, we average 25 to 50 neighbor points according to their distance to TSS, and the data were then smoothed using GraphPad Prism 5.0.

For gene body methylation, the average of CpG sites located +1000 bp from the start of transcription to the transcription end site was calculated. For GSEA, gene sets were downloaded from the Broad Institute’s MSigDB website [29]. Gene set permutations were used to determine statistical enrichment of the gene sets using the difference of methylation between Twist1-transduced cells (monolayer) and vector cells.

### Chromatin immunoprecipitation-sequence generation and mapping

ChIP-seq experiments performed for H3K4m3 and H3K27me3 produced more than 10 million uniquely mapped tags per chromatin modification. ChIP was performed according to the Abcam protocol [52] with few modifications. Library preparation and sequencing were performed on an Illumina/Solexa Genome Analyzer II or Hiseq 2000 in accordance with the manufacturer’s protocols. ChIP-seq reads were aligned to the human genome (hg18) using the Illumina Analyzer pipeline.

Unique reads mapped to a single genomic location were called peaks using the MACS software (version 1.3.7.1) for H3K4me3 marks (the window was 400 bp, and the *P*-value cutoff = 1e^-5^) [53].

For peak calling of H3K27me3, SICER (version 1.03) was used to detect peaks and enriched domains as the peaks were large and not as sharp as for H3K4me3 [54]. The window size was set as 200 bp as default. The gap size was determined as recommended by Zang *et al*. [54], or at most 2 kb, since the performance worsens as the gap size increases beyond more than 10 times the window size. Following Wang *et al*. [55], the E-value was set at FDR ≤5%, which was estimated as E-value (the expected number of significant domains under the random background) divided by the number of identified candidate domains. The FDR cutoff to further filter out the candidate domains by comparing to control was set as 5%.

Sequencing reads for histone H3 DNA were used as control for MACS and SICER. Annotated RefSeq genes with a peak located at their promoters (−1 kb to +0.5 kb of TSS) were identified as being marked by H3K4me3 or H3K27me3 modifications. For the pathway analysis, GO analysis was done using DAVID [56,57]. DAVID analyses were performed online using parameters of EASE value of <1 × 10–5, count of >10, fold enrichment of >2 and Bonfferroni of <1 × 10–2. For GSEA, gene sets were downloaded from the Broad Institute’s MSigDB website [29]. Gene set permutations were used to determine statistical enrichment of the gene sets using the fold enrichment difference in histone modifications between H3K4me3 and H3K27me3 of mesenchymal cells (Twist1 cells) and vector cells.

To exclude the possibility of technical variations, we performed technical (independent IP) replicates for the ChIP of H3K4me3 and H3K27me3 in HMLE cells transduced with Twist1 and cultured in spheres followed by sequencing. Likewise, we performed a technical replicate for ChIP of H3K27me3 in HMLE vector cells. We obtained high correlations between the technical replicates (r >0.82; Table S10 in Additional file 1), suggesting that our findings were not due to chance. A list of primers used for ChIP-qPCR validation of selected genes is available in Table S11 in Additional file 1.

### RNA-sequence library generation and mapping

RNA extraction from vector cells and Twist1-transduced cells (monolayer and sphere) were done with Trizol reagent (Invitrogen, 15596–026). Library preparation was done using a SOLiD™ Total RNA-seq Kit according to the manufacturer’s protocol (Life Technologies, Carlsbad, CA, USA). Reads sequenced produced by the SOLiD analysis pipeline were aligned with to the National Center for Biotechnology Information BUILD hg19 reference sequence. Short reads were mapped to the human reference genome (hg19) and exon junctions using the ABI Bioscope (version 1.21) pipeline with default parameters. Only the tags that mapped to the hg19 reference at full 35-nucleotide length were used. Reads that aligned to multiple positions were excluded. Tags mapped to RefSeq genes were counted to derive a measure of gene expression. To compare the gene expression values, we reasoned that cell type change associated with EMT could result in a change in the total amount of RNA. We therefore used the most conservative normalization by assuming most genes did not change their expression. This was done by constructing a histogram of expression ratio and by assuming that the maximum of the histogram corresponded to no change in gene expression. When compared to the normalization procedure where the total tags mapped to the genes were assumed to be constant, the differences were less than 10%.

### Data availability

All sequencing data and processed files are available on Gene Expression Omnibus accession number [GEO:GSE53026].

## Abbreviations

bp: base pairs; ChIP-seq: chromatin immunoprecipitation sequencing; CGI: CpG islands; DREAM: digital restriction enzyme analysis of methylation; EMT: epithelial-mesenchymal transition; FDR: false discovery rate; GO: Gene Ontology; GSEA: gene set enrichment analysis; HMLE: human mammary epithelial cells; MS: mammosphere; PcG: Polycomb group proteins; PCR: polymerase chain reaction; PMD: partially methylated domain; qPCR: quantitative polymerase chain reaction; shRNA: short hairpin RNA; TGF-β: transforming growth factor beta; TrxG: Trithorax group proteins; TSS: transcription start site.

## Competing interests

The authors declare that they have no competing interests.

## Authors’ contributions

GGM, JHT, SAM and JPI conceived and designed the experiments. GGM, JHT, TR, SP, MRE, JJ, NJR, HL, TT, AT and PR performed the experiments. GGM, YL, XYZ and SL analyzed the data. GGM, JHT, SAM and JPI wrote the paper. All authors read and approved the final manuscript.

## Authors’ information

GGM and JHT are first co-authors.

SAM and JPI are senior co-authors.

## Supplementary Material

Additional file 1: Table S1 Coverage of sequencing of CpG sites using DREAM. **Table S2.** Characteristics of CpG sites in HMLE vector cells that lose and gain DNA methylation in HMLE cells transduced with Twist1. The minimal number of tags used by CpG site is ≥100 tags. **Table S3.** GSEA of top 10 pathways that have differentially gene body methylation in HMLE Twist cells as compared to HMLE vector cells. **Table S4.** Top GSEA for genes marked by H3K4me3 that display statistically significant fold change enrichment in HMLE Twist cells as compared to HMLE vector cells. **Table S5.** Genes presenting promoter switches for H3K4me3 to H3K27me3 histone marks between HMLE vector and HMLE Twist cells. **Table S6.** Top GSEA for genes marked by H3K27me3 that display statistically significant fold change enrichment in HMLE Twist cells as compared to HMLE vector cells. **Table S7.** Top GSEA for differentially expressed genes between HMLE Twist cells as compared to HMLE vector cells. **Table S8.** Summary of relationship between DNA methylation and histone modifications for selected genes. **Table S9.** Top upstream regulators identified using Ingenuity Pathway Analysis for differentially expressed genes between HMLE Twist cells as compared to HMLE vector cells. **Table S10.** Summary of technical ChIP-seq replicates performed. **Table S11.** List of ChIP-qPCR primers used in the study of H3K4me3 and H3K27me3. Click here for file

Additional file 2: Figure S1 Description of DREAM data. **(A)** DNA methylation level (y-axis) of CpG sites located in CGI (green) and non-CGI (red) according to the distance to the TSS (x-axis). Note the higher level of gene body methylation in comparison to the methylation levels of upstream regions. **(B)** DNA methylation level (y-axis) of CpG sites located in PMDs (red) and outside PMDs (green) according to the distance to TSS (x-axis). **(C)** Average DNA methylation levels (y-axis) of CpG sites located in PMDs of HMLE vector cells (blue) and HMLE Twist cells (red). x-axis represents distance of CpG sites to TSS. Note global DNA demethylation of PMDs in mesenchymal cells coupled with increased methylation at promoters. **(D)** Average DNA methylation levels (y-axis) of CpG sites located outside PMDs in HMLE vector cells (blue) and HMLE Twist cells (red). x-axis represents distance of CpG sites to TSS. Note the absence of global DNA demethylation as it is the case for DNA methylation within PMDs. Click here for file

Additional file 3: Figure S2 Average DNA methylation levels in different genomic locations. **(A)** Average DNA methylation level of CpG sites located within/outside partially methylated domains (PMDs) and/or repetitive elements. ns stands for not significant. * *P* <0.05. **(B)** Average DNA methylation level of CpG sites located within/outside PMDs and/or CpG islands. **(C)** Average DNA methylation level of CpG sites located within/outside PMDs and/or lamina associated domains. **(D)** Average DNA methylation level of CpG sites located in repetitive elements of HMLE vector cells (blue) and in HMLE Twist cells cultured in monolayer. ***P <0.0001. Click here for file

Additional file 4: Figure S3 Histone switches of highly up-regulated and down-regulated genes in HMLE Twist cells as compared to HMLE vector cells. The majority of genes that become up-regulated in mesenchymal cells and were pre-marked by H3K27me3 in vector cells switched to H3K4me3 in HMLE Twist cells. Conversely, the majority of genes that become down-regulated in mesenchymal cells and were pre-marked by H3K4me3 in vector cells switched to H3K27me3. Click here for file

Additional file 5: Figure S4 ChIP-qPCR results for H3K4me3 and H3K27me3 in 17 loci in HMLE vector cells, and HMLE Twist-, Snail- and TGF-β1-mediated EMT. Click here for file

Additional file 6: Figure S5 Pearson correlation of ChIP-qPCR results between HMLE Twist, Snail and TGF-β1 cells. Click here for file

Additional file 7: Figure S6 DNA methylation changes using bisulfite sequencing in seven selected gene promoters following Twist-, Snail- and TGF-β1-mediated EMT. Click here for file

Additional file 8: Figure S7 DNA methylation changes using bisulfite sequencing in seven selected gene promoters following Twist-, Snail- and TGF-β1-mediated EMT. Click here for file

Additional file 9: Figure S8 DNA methylation changes using bisulfite sequencing in seven selected gene promoters following Twist-, Snail- and TGF-β1-mediated EMT. Click here for file

Additional file 10: Figure S9 DNA methylation and histone modifications in HMLE Twist cells cultured as spheres. **(A)** Box-plot of gene expression fold change (the bars represent 10% and 90% extreme) for genes switching in HMLE vector cells from H3K4me3, H3K27me3, bivalent or neither marks to other histone marks in HMLE Twist cells cultured as spheres. **(B)** Correlation of methylation level of CpG sites detected by DREAM in HMLE Twist cells cultured in a monolayer (2D) (x-axis) and as spheres (MS) (y-axis). Green, CpG sites located in CGI; red, CpG sites located outside CGI. **(C)** Switches of histone marks between HMLE Twist cells cultured in a monolayer and as spheres. Click here for file

Additional file 11: Figure S10 Expression of EMT-related transcription factors after EZH2 knockdown. qRT-PCR performed for *FOXC2***(A)**, *SNAIL***(B)** and *ZEB1***(C)** using RNA extracted from control and shEZH2 cells showed that the expression level of those EMT-related transcription factors remain unchanged. Click here for file

Additional file 12: Figure S11 EZH1 knockdown in HMLE Twist cells. **(A)** qRT-PCR expression of EZH1 in the indicated cell lines. **(B)** Knockdown of EZH1 mediated by two independent shRNAs. EZH1 mRNA expression was quantified by RT-PCR in the indicated cell lines. **(C)** Control and shEZH1 cells were grown in MS-promoting conditions and spheres greater in size than 50 μm were counted after 10 days. A Student’s t-test was performed. Click here for file

Additional file 13: Figure S12 Twist expression generated cells with consistent levels of vimentin and Foxc2 expression. HMLE vector and HMLE Twist cells were stained for vimentin (a) and for Foxc2 (b) along with a DAPI co-stain. Click here for file

Additional file 14: Figure S13 Twist, Snail and TGF-β1 expression generated cells with consistently elevated levels of vimentin. HMLE vector, HMLE Snail, HMLE Twist and HMLE TGF-β1 cells were stained for vimentin along with a DAPI co-stain. The yellow arrow indicates a cell without appreciable expression of vimentin. Click here for file
