# Supplementary material for: Architecture of epigenetic reprogramming following Twist1-mediated epithelial-mesenchymal transition
Source: Genome Biol. 2013 Dec 24;14(12):R144. doi: 10.1186/gb-2013-14-12-r144 (PMC4053791; doi:10.1186/gb-2013-14-12-r144)
Supplement: Additional file 1: Table S1 — Coverage of sequencing of CpG sites using DREAM. Table S2. Characteristics of CpG sites in HMLE vector cells that lose and gain DNA methylation in HMLE cells transduced with Twist1. The minimal number of tags used by CpG site is ≥100 tags. Table S3. GSEA of top 10 pathways that have differentially gene body methylation in HMLE Twist cells as compared to HMLE vector cells. Table S4. Top GSEA for genes marked by H3K4me3 that display statistically significant fold change enrichment in HMLE Twist cells as compared to HMLE vector cells. Table S5. Genes presenting promoter switches for H3K4me3 to H3K27me3 histone marks between HMLE vector and HMLE Twist cells. Table S6. Top GSEA for genes marked by H3K27me3 that display statistically significant fold change enrichment in HMLE Twist cells as compared to HMLE vector cells. Table S7. Top GSEA for differentially expressed genes between HMLE Twist cells as compared to HMLE vector cells. Table S8. Summary of relationship between DNA methylation and histone modifications for selected genes. Table S9. Top upstream regulators identified using Ingenuity Pathway Analysis for differentially expressed genes between HMLE Twist cells as compared to HMLE vector cells. Table S10. Summary of technical ChIP-seq replicates performed. Table S11. List of ChIP-qPCR primers used in the study of H3K4me3 and H3K27me3. [file gb-2013-14-12-r144-S1.doc]

**Supplementary data**

**Table 1: Coverage of sequencing of CpG sites using DREAM**

|  | **HMLE GFP** | **HMLE Parental** | **HMLE Twist-2D** | **HMLE Twist-3D** |
| --- | --- | --- | --- | --- |
| **Number of tags**  **Methylated**  **Unmethylated** | 8,481,478  23,628,413 | 8,976,057  29,457,650 | 9,517,853  27,964,669 | 9,626,025  23,770,157 |
| **Total number of tags** | 32,109,891 | 38,443,707 | 37,482,522 | 33,396,182 |
| **Number of SmaI/XmaI sites ≥ 1 tag** | 155,309 | 120,502 | 140,907 | 138,233 |
| **Number of SmaI/XmaI sites covered ≥ 10 tags** | 101,248 | 37,075 | 40,180 | 44,180 |
| **Number of SmaI/XmaI sites covered ≥ 100 tags** | 38,603  Average= 701 (100-197279) | 29,166  Average=1286 (100-172794) | 29,276  Average=1244 (100-238096) | 31,218  Average=1005 (100-302550) |
| **Nb of promoters covered** | **5791**  **(average=2.5 CpG sites/promoter)** | **4851**  **(average=2.7 CpG sites/promoter)** | **4876**  **(average=2.6 CpG sites/promoter)** | **5083**  **(average=2.6 CpG sites/promoter)** |

**Table 2: Characteristics of CpG sites in HMLE parental cells that lose and gain DNA methylation in HMLE cells transfected with Twist1. The minimal number of tags used by CpG site is ≥100tags.**

| **Characteristics** | | **Overall Number of CpG sites** | **Number of CpG sites with a methylation level in HMLE parental cells ≥70%** | **Number of CpG sites with loss of at least 20% methylation in HMLE cells transfected with Twist1** | **p-value** | **Number of CpG sites with a methylation level in HMLE parental cells ≤10%** | **Number of CpG sites with gain of at least 20% methylation in HMLE cells transfected with Twist1** | **p-value** |
| --- | --- | --- | --- | --- | --- | --- | --- | --- |
| **CpG islands** | Yes  No | 15322  11191 | 861  4042 | 125 (14.5%)  787 (19.5%) | 0.0007 | 12680  3502 | 187 (1.5%)  139 (4%) | <0.0001 |
| **Repeats** | Yes  No | 3289  23224 | 663  4240 | 138 (20.8%)  773 (18.2%) | ns | 2023  14159 | 33 (1.6%)  293 (2.1%) | ns |
| **Locations** | Promoter  Genebody  Upstream or downstream | 11670  5394  9449 | 232  2001  2670 | 50 (21.5%)  335 (16.7%)  526 (19.7%) | 0.02 | 10473  1824  3885 | 113 (1.1%)  51 (2.8%)  162 (4.2%) | <0.0001 |
| **PMD** | Yes  No | 6649  19864 | 693  4210 | 217 (31.3%)  694 (16.5%) | <0.0001 | 4088  12094 | 160 (3.9%)  166 (1.4%) | <0.0001 |
| **LAD** | Yes  No | 3525  22988 | 479  4424 | 132 (27.6%)  779 (17.7%) | <0.0001 | 2179  14003 | 87 (4%)  239 (1.7%) | <0.0001 |

For this analysis only methylated CpG sites with ≥70% methylation in HMLE parental cells and that lose ≥20% methylation in HMLE cells transfected with Twist1 were taken in account. In addition, only CpG sites with less than 10% methylation in HMLE parental cells and that gain ≥20% methylation in HMLE cells transfected with Twist1 were also taken in account. Abbreviations: PMD, partially methylated domains; LAD: Lamina-associated domains.

| GS<br> follow link to MSigDB | SIZE | ES | NES | NOM p-val | FDR q-val |
| --- | --- | --- | --- | --- | --- |
| TOYOTA_TARGETS_OF_MIR34B_AND_MIR34C | 36 | -0.72 | -1.81 | 0.00 | 0.05 |
| DAZARD_UV_RESPONSE_CLUSTER_G6 | 26 | -0.74 | -1.78 | 0.00 | 0.05 |
| ONDER_CDH1_TARGETS_2_DN | 65 | -0.62 | -1.74 | 0.00 | 0.08 |
| REACTOME_TRANSMEMBRANE_TRANSPORT_OF_  SMALL_MOLECULES | 34 | -0.67 | -1.66 | 0.00 | 0.27 |
| REACTOME_SLC_MEDIATED_TRANSMEMBRANE_TRANSPORT | 31 | -0.66 | -1.64 | 0.01 | 0.31 |
| SHEN_SMARCA2_TARGETS_DN | 60 | -0.60 | -1.63 | 0.00 | 0.30 |
| DAZARD_RESPONSE_TO_UV_NHEK_DN | 40 | -0.62 | -1.62 | 0.00 | 0.31 |
| CHARAFE_BREAST_CANCER_LUMINAL_VS_BASAL_DN | 68 | -0.57 | -1.61 | 0.00 | 0.30 |
| HAHTOLA_MYCOSIS_FUNGOIDES_SKIN_UP | 20 | -0.70 | -1.61 | 0.01 | 0.28 |
| WINTER_HYPOXIA_METAGENE | 33 | -0.64 | -1.58 | 0.01 | 0.35 |

**Table 3: Gene set enrichment analysis of top 10 pathways that have differentially gene body methylation in HMLE Twist1-cells as compared to HMLE parental cells**

**Table 4: Top gene set enrichment analysis for genes marked by H3K4me3 and which display statistically significant fold change enrichment in** HMLE Twist1-cells as compared to HMLE parental cells

| GS<br> follow link to MSigDB | ES | NES | NOM p-val | FDR q-val |
| --- | --- | --- | --- | --- |
| KEGG_GRAFT_VERSUS_HOST_DISEASE | 0.873 | 1.621 | 0.000 | 0.013 |
| WEBER_METHYLATED_ICP_IN_FIBROBLAST | 0.874 | 1.508 | 0.001 | 0.152 |
| KEGG_AUTOIMMUNE_THYROID_DISEASE | 0.786 | 1.489 | 0.000 | 0.158 |
| REACTOME_TRANSLOCATION_OF_ZAP70_TO_IMMUNOLOGICAL_SYNAPSE | -1.000 | -2.624 | 0.000 | 0.000 |
| REACTOME_PHOSPHORYLATION_OF_CD3_AND_TCR_ZETA_CHAINS | -0.908 | -2.502 | 0.000 | 0.001 |
| REACTOME_XENOBIOTICS | -0.896 | -2.145 | 0.024 | 0.011 |
| REACTOME_FORMATION_OF_FIBRIN_CLOT_CLOTTING_CASCADE | -0.752 | -2.126 | 0.000 | 0.008 |
| PYEON_CANCER_HEAD_AND_NECK_VS_CERVICAL_DN | -0.743 | -2.090 | 0.000 | 0.008 |
| REACTOME_INTRINSIC_PATHWAY | -0.777 | -1.908 | 0.000 | 0.021 |
| AIGNER_ZEB1_TARGETS | -0.614 | -1.803 | 0.000 | 0.034 |
| YANG_BREAST_CANCER_ESR1_BULK_DN | -0.665 | -1.692 | 0.000 | 0.049 |
| ROY_WOUND_BLOOD_VESSEL_DN | -0.607 | -1.604 | 0.032 | 0.070 |
| MUELLER_METHYLATED_IN_GLIOBLASTOMA | -0.459 | -1.414 | 0.000 | 0.179 |
| REACTOME_NA_CL_DEPENDENT_NEUROTRANSMITTER_TRANSPORTERS | -0.541 | -1.391 | 0.063 | 0.182 |
| ZHAN_MULTIPLE_MYELOMA_SPIKED | -0.534 | -1.326 | 0.143 | 0.239 |

Table 5: genes presenting promoter switches for H3K4me3 to H3K27me3 histone marks between HMLE parental and HMLE Twist1-induced cells

| Genes switching from H3K4me3 in HMLE parental cells to H3K27me3 in HMLE Twist1-induced cells | Genes switching from H3K27me3 in HMLE parental cells to H3K4me3 in HMLE Twist1-induced cells |
| --- | --- |
| AMPD3 | ABR |
| BEX4 | ADAM33 |
| BMP2 | ADAMTS5 |
| BMPR1B | ADNP2 |
| C1orf106 | AFF3 |
| CCDC64 | AGTR1 |
| CCNJL | ARID3C |
| CDH1 | ATOH8 |
| CHMP4C | BEAN |
| CWH43 | BFSP1 |
| CYP2R1 | BGN |
| DDIT4L | BNC2 |
| DSC2 | BRSK2 |
| EPHX2 | BSN |
| ESRP1 | C14orf132 |
| ESYT3 | C17orf102 |
| EXPH5 | C17orf72 |
| FA2H | C18orf1 |
| FAM110C | C1orf133 |
| FAM171A1 | C20orf201 |
| FAM189A2 | C3orf18 |
| FAM83B | C3orf55 |
| FGFR2 | CADM3 |
| GOLGA7B | CBLN2 |
| GRB14 | CBX6 |
| GRHL2 | CCDC22 |
| HOOK1 | CDKN1C |
| HPCAL4 | CMPK2 |
| HS3ST2 | CNRIP1 |
| KALRN | COL6A2 |
| KANK4 | CRHBP |
| KCNK1 | DACT3 |
| KIAA1244 | DAGLA |
| KIAA1804 | DGKI |
| KLK5 | DKFZp779M0652 |
| KLK8 | DKK2 |
| KRT5 | DOCK10 |
| LAD1 | DUSP15 |
| LGR6 | EBF1 |
| LHX1 | FAM78A |
| LRAT | FBLN7 |
| MAF | FBXL15 |
| MAL2 | FBXL7 |
| MDFI | FERD3L |
| MYO5B | FLJ36777 |
| MYO5C | FMN2 |
| NDRG2 | FUCA1 |
| NEURL1B | GCNT2 |
| NMU | GHR |
| NPNT | GNAO1 |
| NT5C1A | GNASAS |
| OVOL1 | GPR173 |
| OVOL2 | GRHL1 |
| P2RY1 | GRID1 |
| PCDHAC1 | GYPC |
| PDLIM3 | HTR1B |
| PLD5 | ID2 |
| PPP1R14C | IFITM2 |
| QRFPR | IGFBP1 |
| RAPGEF5 | INSRR |
| RASSF10 | ITGA7 |
| RNF128 | ITPKA |
| SELV | KIAA1755 |
| SGPP2 | LOC100190938 |
| SH3RF2 | LOC283856 |
| SLC22A3 | LOC728392 |
| SLC35F3 | LRFN3 |
| SLC9A2 | LYPD6 |
| SLFN13 | MAGI2 |
| STK33 | MAP1B |
| SULT1A1 | MAP2K3 |
| SYK | MEG3 |
| TFCP2L1 | MIAT |
| TLR2 | NCALD |
| WNT10A | NID1 |
|  | NPTX2 |
|  | NR2F1 |
|  | NTRK1 |
|  | OLFML2B |
|  | OSR1 |
|  | PAPLN |
|  | PCDHGA2 |
|  | PDE2A |
|  | PDE4D |
|  | PDE9A |
|  | PDGFRA |
|  | PDZRN3 |
|  | PLAC9 |
|  | PLCXD3 |
|  | PMP22 |
|  | PPFIA2 |
|  | PRDM2 |
|  | PREX1 |
|  | PRKAR2B |
|  | PRUNE2 |
|  | PSD |
|  | PTCHD2 |
|  | PTGIS |
|  | PTPRD |
|  | PTPRS |
|  | PTX3 |
|  | PYGO1 |
|  | RAMP2 |
|  | RHBDL3 |
|  | RICH2 |
|  | RNF150 |
|  | RNF157 |
|  | RTN4RL2 |
|  | SCARF2 |
|  | SDC2 |
|  | SERTAD4 |
|  | SIX2 |
|  | SLC12A7 |
|  | SLC16A6 |
|  | SLC4A8 |
|  | SLFN11 |
|  | SLITRK1 |
|  | SMARCD3 |
|  | SPOCK1 |
|  | STXBP6 |
|  | SYNGR1 |
|  | SYPL2 |
|  | SYT10 |
|  | TBX18 |
|  | TBX2 |
|  | TERT |
|  | TMEM132E |
|  | TNFRSF1B |
|  | TRO |
|  | TTC28 |
|  | TTLL9 |
|  | TUBB2B |
|  | USP51 |
|  | VENTX |
|  | WDR86 |
|  | WNT16 |
|  | WNT5A |
|  | ZEB2 |
|  | ZFP28 |
|  | ZFYVE28 |
|  | ZNF256 |
|  | ZNF334 |
|  | ZNF610 |
|  | ZNF804A |

**Table 6**: Top gene set enrichment analysis for genes marked by H3K27me3 and which display statistically significant fold change enrichment in HMLE Twist1-cells as compared to HMLE parental cells

| GS<br> follow link to MSigDB | ES | NES | NOM p-val | FDR q-val |
| --- | --- | --- | --- | --- |
| BOYAULT_LIVER_CANCER_SUBCLASS_G3_UP | 0.978 | 1.996 | 0.000 | 0 |
| KOBAYASHI_EGFR_SIGNALING_24HR_DN | 0.962 | 1.991 | 0.000 | 0 |
| CHARAFE_BREAST_CANCER_LUMINAL_VS_BASAL_DN | 0.916 | 1.991 | 0.000 | 0 |
| REACTOME_FORMATION_AND_MATURATION_OF_MRNA_TRANSCRIPT | 0.979 | 1.933 | 0.000 | 0 |
| SOTIRIOU_BREAST_CANCER_GRADE_1_VS_3_UP | 0.983 | 1.924 | 0.000 | 0 |
| REACTOME_ELONGATION_AND_PROCESSING_OF_CAPPED_TRANSCRIPTS | 0.979 | 1.919 | 0.000 | 0 |
| GARGALOVIC_RESPONSE_TO_OXIDIZED_PHOSPHOLIPIDS_BLUE_UP | 0.960 | 1.913 | 0.000 | 0 |
| KEGG_SPLICEOSOME | 0.977 | 1.909 | 0.000 | 0 |
| ONDER_CDH1_TARGETS_2_DN | 0.877 | 1.904 | 0.000 | 0 |
| REACTOME_PROCESSING_OF_CAPPED_INTRON_CONTAINING_PRE_MRNA | 0.979 | 1.904 | 0.000 | 0 |
| PYEON_CANCER_HEAD_AND_NECK_VS_CERVICAL_UP | 0.949 | 1.900 | 0.000 | 0 |
| REACTOME_DNA_REPAIR | 0.979 | 1.851 | 0.000 | 1.26E-04 |
| REACTOME_MRNA_SPLICING | 0.979 | 1.850 | 0.000 | 1.16E-04 |
| CHIANG_LIVER_CANCER_SUBCLASS_UNANNOTATED_DN | 0.939 | 1.847 | 0.000 | 1.08E-04 |
| REACTOME_APOPTOSIS | 0.938 | 1.844 | 0.000 | 1.01E-04 |
| REACTOME_LATE_PHASE_OF_HIV_LIFE_CYCLE | 0.979 | 1.842 | 0.000 | 9.43E-05 |
| REACTOME_METABOLISM_OF_RNA | 0.985 | 1.838 | 0.000 | 8.88E-05 |
| TOOKER_RESPONSE_TO_BEXAROTENE_UP | 0.943 | 1.834 | 0.000 | 8.39E-05 |
| PUJANA_BREAST_CANCER_LIT_INT_NETWORK | 0.965 | 1.825 | 0.000 | 7.94E-05 |
| NOUZOVA_TRETINOIN_AND_H4_ACETYLATION | 0.955 | 1.823 | 0.000 | 7.55E-05 |
| ROSTY_CERVICAL_CANCER_PROLIFERATION_CLUSTER | -1.000 | -1.775 | 0.000 | 0 |
| YAO_TEMPORAL_RESPONSE_TO_PROGESTERONE_CLUSTER_17 | -0.945 | -1.709 | 0.000 | 0 |
| HOFFMANN_LARGE_TO_SMALL_PRE_BII_LYMPHOCYTE_UP | -1.000 | -1.706 | 0.000 | 0 |
| LAIHO_COLORECTAL_CANCER_SERRATED_UP | -0.955 | -1.676 | 0.000 | 0 |
| REACTOME_MITOTIC_PROMETAPHASE | -0.982 | -1.665 | 0.000 | 0 |
| MOREAUX_MULTIPLE_MYELOMA_BY_TACI_DN | -0.935 | -1.661 | 0.000 | 0 |
| REACTOME_RNA_POLYMERASE_I_PROMOTER_CLEARANCE | -0.989 | -1.652 | 0.000 | 7.02E-04 |
| WINNEPENNINCKX_MELANOMA_METASTASIS_UP | -0.916 | -1.643 | 0.000 | 9.62E-04 |
| KEGG_UBIQUITIN_MEDIATED_PROTEOLYSIS | -0.933 | -1.642 | 0.001 | 8.55E-04 |
| RUIZ_TNC_TARGETS_DN | -0.928 | -1.638 | 0.000 | 7.70E-04 |
| KEGG_RIG_I_LIKE_RECEPTOR_SIGNALING_PATHWAY | -1.000 | -1.626 | 0.000 | 0.001655503 |
| KYNG_DNA_DAMAGE_UP | -0.934 | -1.616 | 0.000 | 0.00251159 |
| HESS_TARGETS_OF_HOXA9_AND_MEIS1_UP | -1.000 | -1.612 | 0.000 | 0.00264221 |
| KEGG_ANTIGEN_PROCESSING_AND_PRESENTATION | -0.949 | -1.610 | 0.000 | 0.002908799 |
| REACTOME_FORMATION_OF_A_POOL_OF_FREE_40S_SUBUNITS | -0.931 | -1.607 | 0.000 | 0.003324571 |
| CROONQUIST_NRAS_SIGNALING_DN | -1.000 | -1.602 | 0.000 | 0.004167598 |
| WAKASUGI_HAVE_ZNF143_BINDING_SITES | -1.000 | -1.602 | 0.000 | 0.003922446 |
| LAU_APOPTOSIS_CDKN2A_UP | -1.000 | -1.601 | 0.000 | 0.003861036 |
| RHODES_UNDIFFERENTIATED_CANCER | -1.000 | -1.598 | 0.000 | 0.004212708 |
| KEGG_PANCREATIC_CANCER | -0.960 | -1.591 | 0.000 | 0.006074989 |

Table 7: Top gene set enrichment analysis for differentially expressed genes between HMLE Twist1-cells as compared to HMLE parental cells

| GS<br> follow link to MSigDB | ES | NES | NOM p-val | FDR q-val |
| --- | --- | --- | --- | --- |
| BROWNE_INTERFERON_RESPONSIVE_GENES | 0.78 | 2.43 | 0 | 0 |
| DAUER_STAT3_TARGETS_DN | 0.85 | 2.42 | 0 | 0 |
| MOSERLE_IFNA_RESPONSE | 0.89 | 2.37 | 0 | 0 |
| TAKEDA_TARGETS_OF_NUP98_HOXA9_FUSION_3D_UP | 0.65 | 2.32 | 0 | 0 |
| SANA_TNF_SIGNALING_UP | 0.70 | 2.22 | 0 | 0 |
| SEITZ_NEOPLASTIC_TRANSFORMATION_BY_8P_DELETION_UP | 0.69 | 2.20 | 0 | 0 |
| SANA_RESPONSE_TO_IFNG_UP | 0.70 | 2.20 | 0 | 0 |
| BENNETT_SYSTEMIC_LUPUS_ERYTHEMATOSUS | 0.86 | 2.19 | 0 | 0 |
| ZHANG_INTERFERON_RESPONSE | 0.88 | 2.19 | 0 | 0 |
| TAKEDA_TARGETS_OF_NUP98_HOXA9_FUSION_8D_UP | 0.61 | 2.18 | 0 | 0 |
| FARMER_BREAST_CANCER_CLUSTER_1 | 0.77 | 2.17 | 0 | 0 |
| EINAV_INTERFERON_SIGNATURE_IN_CANCER | 0.81 | 2.13 | 0 | 8.33E-05 |
| TAKEDA_TARGETS_OF_NUP98_HOXA9_FUSION_10D_UP | 0.58 | 2.09 | 0 | 1.50E-04 |
| TAKEDA_TARGETS_OF_NUP98_HOXA9_FUSION_16D_UP | 0.57 | 2.05 | 0 | 4.90E-04 |
| KRASNOSELSKAYA_ILF3_TARGETS_UP | 0.76 | 2.05 | 0 | 4.57E-04 |
| LIANG_SILENCED_BY_METHYLATION_2 | 0.75 | 2.05 | 0 | 4.28E-04 |
| MAHADEVAN_RESPONSE_TO_MP470_UP | 0.84 | 2.03 | 0 | 5.20E-04 |
| CHIANG_LIVER_CANCER_SUBCLASS_INTERFERON_UP | 0.75 | 2.01 | 0 | 8.16E-04 |
| XU_AKT1_TARGETS_6HR | 0.77 | 2.00 | 0 | 9.28E-04 |
| UROSEVIC_RESPONSE_TO_IMIQUIMOD | 0.89 | 1.99 | 0 | 8.81E-04 |
| ROSTY_CERVICAL_CANCER_PROLIFERATION_CLUSTER | -0.67 | -2.17 | 0 | 0 |
| REACTOME_RNA_POLYMERASE_I_PROMOTER_OPENING | -0.74 | -2.08 | 0 | 0 |
| SOTIRIOU_BREAST_CANCER_GRADE_1_VS_3_UP | -0.64 | -2.06 | 0 | 0 |
| REACTOME_RNA_POLYMERASE_I_PROMOTER_CLEARANCE | -0.68 | -2.06 | 0 | 0 |
| KOBAYASHI_EGFR_SIGNALING_24HR_DN | -0.60 | -2.03 | 0 | 0 |
| REACTOME_PACKAGING_OF_TELOMERE_ENDS | -0.72 | -2.00 | 0 | 1.67E-04 |
| KANG_DOXORUBICIN_RESISTANCE_UP | -0.70 | -1.97 | 0 | 8.56E-04 |
| WINNEPENNINCKX_MELANOMA_METASTASIS_UP | -0.59 | -1.95 | 0 | 0.001628177 |
| REACTOME_TELOMERE_MAINTENANCE | -0.65 | -1.94 | 0 | 0.001785544 |
| HORIUCHI_WTAP_TARGETS_DN | -0.55 | -1.93 | 0 | 0.002505135 |
| NAKAYAMA_SOFT_TISSUE_TUMORS_PCA2_UP | -0.63 | -1.92 | 0 | 0.002459926 |
| WHITEFORD_PEDIATRIC_CANCER_MARKERS | -0.63 | -1.90 | 0 | 0.00284138 |
| CROONQUIST_NRAS_SIGNALING_DN | -0.66 | -1.89 | 0 | 0.004326128 |
| ZHAN_MULTIPLE_MYELOMA_PR_UP | -0.70 | -1.88 | 0 | 0.004520109 |
| FARMER_BREAST_CANCER_CLUSTER_2 | -0.73 | -1.87 | 0 | 0.00495761 |
| REACTOME_RNA_POLYMERASE_I_III_AND_MITOCHONDRIAL_TRANSCRIPTION | -0.60 | -1.86 | 0 | 0.005655071 |
| WONG_EMBRYONIC_STEM_CELL_CORE | -0.53 | -1.86 | 0 | 0.006565704 |
| FURUKAWA_DUSP6_TARGETS_PCI35_DN | -0.64 | -1.85 | 0 | 0.007775272 |
| BASAKI_YBX1_TARGETS_UP | -0.53 | -1.83 | 0 | 0.009541415 |

**Table 8: Summary of relation between DNA methylation and histone modifications for selected genes**

| **Gene name** | **Promoter DNA methylation (%) in HMLE parental cells*** | **Promoter DNA methylation (%) in HMLE Twist1 cells*** | **Genes switching from H3K4me3 mark in HMLE parental cells to H3K27me3 mark in HMLE Twist1 cells°** |
| --- | --- | --- | --- |
| **OVOL1** | **0.83** | **18.84** | **Yes** |
| **SGPP2** | **11.93** | **42.44** | **Yes** |
| **FGFR2** | **3.12** | **27.60** | **Yes** |
| **FAM110C** | **10.28** | **35.76** | **Yes** |
| **SYK** | **0.13** | **24.94** | **Yes** |
| **NPNT** | **1.5** | **41.67** | **Yes** |
| **TRHDE** | **0.83** | **4.78** | **Yes** |

*** Promoter DNA Methylation of selected genes was performed using bisulfite sequencing**

**° H3K27me3 was assessed using ChIP-qPCR**

**Table 9**: Top upstream regulators identified using Ingenuity Pathway Analysis for differentially expressed genes between HMLE Twist1-cells as compared to HMLE parental cells

| Upstream Regulator | Fold Change | Molecule Type | Predicted Activation State | Activation z-score | p-value of overlap |
| --- | --- | --- | --- | --- | --- |
| estrogen receptor |  | group | Inhibited | -3.75 | 5.60E-18 |
| IL1A | -4.738 | cytokine | Inhibited | -4.79 | 1.81E-13 |
| TNF | -4.886 | cytokine | Inhibited | -6.736 | 7.81E-13 |
| EZH2 |  | transcription regulator | Inhibited | -3.535 | 8.25E-11 |
| IL1B | -5.52 | cytokine | Inhibited | -4.662 | 1.03E-10 |
| NFkB (complex) |  | complex | Inhibited | -5.184 | 1.44E-08 |
| RELA |  | transcription regulator | Inhibited | -2.917 | 2.50E-08 |
| JUN | -0.202 | transcription regulator | Inhibited | -2.961 | 2.42E-07 |
| PPRC1 | -0.251 | transcription regulator | Inhibited | -2.811 | 1.04E-06 |
| TREM1 | -0.664 | transmembrane receptor | Inhibited | -2.317 | 1.79E-06 |
| EGFR |  | kinase | Inhibited | -3.716 | 2.51E-06 |
| CSF2 | -4.613 | cytokine | Inhibited | -2.376 | 2.55E-06 |
| P38 MAPK |  | group | Inhibited | -2.702 | 3.39E-06 |
| IgG |  | complex | Activated | 3.168 | 4.24E-06 |
| SPDEF | 4.296 | transcription regulator | Inhibited | -2.463 | 8.42E-06 |
| TLR4 | 2.539 | transmembrane receptor | Inhibited | -2.052 | 1.92E-05 |
| EGR1 | 0.606 | transcription regulator | Inhibited | -2.934 | 3.24E-05 |
| lymphotoxin-alpha1-beta2 |  | complex | Inhibited | -2.538 | 6.85E-05 |
| WISP2 | 0.631 | growth factor | Inhibited | -3.286 | 2.28E-04 |
| JAG2 | -3.667 | growth factor | Activated | 3.902 | 2.84E-04 |
| ESR2 |  | ligand-dependent nuclear receptor | Inhibited | -2.027 | 3.14E-04 |
| TP63 | -5.866 | transcription regulator | Inhibited | -3.522 | 3.82E-04 |
| IFNG | 0 | cytokine | Inhibited | -3.457 | 5.91E-04 |
| ZEB1 | 3.738 | transcription regulator | Activated | 2.582 | 9.19E-04 |
| ERK |  | group | Inhibited | -3.036 | 1.06E-03 |
| MAP3K7 |  | kinase | Activated | 2.5 | 1.76E-03 |
| IL5 | -1.379 | cytokine | Inhibited | -2.295 | 2.06E-03 |
| TLR1 | -0.44 | other | Inhibited | -2.138 | 2.11E-03 |
| SMAD3 |  | transcription regulator | Activated | 2.431 | 2.44E-03 |
| S100A7 | -5.498 | other | Inhibited | -2.236 | 2.95E-03 |
| SKI | 0.395 | transcription regulator | Inhibited | -2 | 2.95E-03 |
| IL18 | -2.202 | cytokine | Inhibited | -2.72 | 3.54E-03 |
| TNFSF12 | 1.814 | cytokine | Inhibited | -2.615 | 5.48E-03 |
| TAB1 |  | enzyme | Activated | 3.051 | 6.26E-03 |
| PGR |  | ligand-dependent nuclear receptor | Inhibited | -2.719 | 7.62E-03 |
| NAMPT | -0.028 | cytokine | Inhibited | -2.804 | 8.39E-03 |
| PTGS2 | -1.19 | enzyme | Inhibited | -2.164 | 9.43E-03 |
| BTK | 3.839 | kinase | Inhibited | -2 | 9.47E-03 |
| ERBB2 |  | kinase | Inhibited | -3.681 | 1.08E-02 |
| miR-155-5p (miRNAs w/seed UAAUGCU) |  | mature microRNA | Activated | 2.434 | 1.14E-02 |
| PF4 | 0 | cytokine | Inhibited | -2.03 | 1.22E-02 |
| Cdk |  | group | Inhibited | -2.213 | 1.31E-02 |
| TNFRSF18 |  | transmembrane receptor | Activated | 2.236 | 1.31E-02 |
| KIAA1524 | 0.092 | other | Activated | 2.393 | 1.31E-02 |
| PLG |  | peptidase | Inhibited | -2.053 | 1.33E-02 |
| FCER1G | 0.494 | transmembrane receptor | Inhibited | -2.449 | 1.39E-02 |
| NLRP12 | -1.379 | other | Activated | 2.415 | 1.39E-02 |
| TLR2 | -4.934 | transmembrane receptor | Inhibited | -2.136 | 1.50E-02 |
| MGEA5 |  | enzyme | Inhibited | -2.4 | 2.02E-02 |
| ZBTB16 | 2.645 | transcription regulator | Activated | 2.158 | 2.32E-02 |
| MYD88 |  | other | Inhibited | -2.382 | 2.76E-02 |
| Immunoglobulin |  | complex | Inhibited | -3.036 | 2.81E-02 |
| CAMP | -4.899 | other | Inhibited | -2.57 | 2.90E-02 |
| TNFSF14 | -4.089 | cytokine | Inhibited | -2.4 | 3.07E-02 |
| TICAM1 | -0.162 | other | Inhibited | -2.236 | 3.07E-02 |
| NOD2 | -4.551 | other | Inhibited | -2.219 | 3.07E-02 |
| LILRB4 |  | other | Activated | 2.165 | 3.41E-02 |
| IL37 | -3.946 | cytokine | Activated | 2.2 | 3.41E-02 |
| IL33 | -3.407 | cytokine | Inhibited | -2.381 | 3.50E-02 |
| SNAI2 | -0.012 | other | Activated | 2.348 | 3.95E-02 |
| SELPLG |  | other | Inhibited | -2.673 | 4.04E-02 |
| CD3 |  | complex | Activated | 2.849 | 4.98E-02 |

**Table 10: Summary of technical ChIP-seq replicates performed**

|  | Parental replicate | Twist1 induced cells (monolayer) (2D) replicate | Twist1 induced cells (Sphere) (3D) replicate |
| --- | --- | --- | --- |
| H3K27me3 | Yes (R= 0.83) | No | Yes (R=0.87) |
| H3K4me3 | No | No | Yes (R=0.930) |

* Libraries were sequenced in Illumina Genome Analyzer 2 and Illumina Hiseq 2000 and compared.

R represents the spearman correlation values

**Table 11: List of ChIP-qPCR primers used in the study of H3K4me3 and H3K27me3**

| ***Primer*** | ***Sequence*** |
| --- | --- |
| 5'ESRP1chr8:95720200-95724799 | **CGGATTACTTGGTGGTGCTT** |
| 5'CDH1chr16:67323400-67330199 | **CAAGGCTTGCTGGGTAGAAG** |
| 5'OVOL2chr20:17978800-17987999 | **AGCGGATACCTTTGAGCAGA** |
| 5'DSC2chr18:26932600-26936799 | **AAGCCTCGCACGTTAAGAAA** |
| 5'KRT5chr12:51188200-51236599 | **CCAGTCTGCTCCAACCTCTC** |
| 5'ZEB2chr2:144988800-144994199 | **CCCATCTCCTTTCTCCTTCC** |
| 5'SMARCD3chr7:150561400-150613799 | **GCCTAGTAGGGGCTCAATCC** |
| 5'CBX6chr22:37598800-37610999 | **GAGCCAGCCAACAGAAGAAC** |
| 5'ID2chr2:8722400-8739999 | **CCTGTAGCACTGCTGTTGGA** |
| 5'ZEB2chr2:144993834-144994244 | **AGTTTTGGCCAGAAATGGTG** |
| 5'SMARCD3chr7:150603809-150605946 | **AAATTTGGCAGCTTGGAGAA** |
| 5'ID2chr2:8739658-8741083 | **CGTGAGGTCCGTTAGGAAAA** |
| 5'CBX6chr22:37596886-37598202 | **CCCAAAACCCTCTTGTTGAA** |
| 5'PDGFRAchr4:54788427-54789434 | **ACCCGCTTTTGCCTTTTATT** |
| 5'ESRP1chr8:95721437-95724616 | **CGGATTACTTGGTGGTGCTT** |
| 5'OVOL1chr11:65311072-65312212 | **GAGCCAATAAAGCGTGAACC** |
| 5'SYKchr9:92604055-92604413 | **CAGCTTCGCTCCTGAACCTA** |
| 5'CYP2R1chr11:14869178-14870388 | **CACCGGCTGGTTATGAGTTT** |
| 5'CDH1chr16:67328383-67330049 | **GAGTCACCCGGTTCCATCTA** |
| 5'FAM110Cchr2:34805-36603 | **TCTCACCACTTCAGCACAGG** |
| 5'SGPP2chr2:222997519-222998089 | **GTAACCATGGGCAGGTGTTC** |
| 3'ESRP1chr8:95722539-95788870 | **CACAAACTTCTTGCCCCATT** |
| 3'CDH1chr16:67328695-67426945 | **GGAGAAGGCTCCGGTATTTC** |
| 3'OVOL2chr20:17952795-17986521 | **TGTCAGACAGGAGGCTTGTG** |
| 3'DSC2chr18:26899939-26936386 | **TTGGGTGAAAAAGGGAACTG** |
| 3'KRT5chr12:51194625-51200510 | **GGACTCAGCTCCACTTTTGC** |
| 3'ZEB2chr2:144988800-144994199 | **GGATGACTCCTCTCCAGCAG** |
| 3'SMARCD3chr7:150561400-150613799 | **CAGGCAGAATGAAGGGGATA** |
| 3'CBX6chr22:37598800-37610999 | **CGGGGGATACATCACAAATC** |
| 3'ID2chr2:8722400-8739999 | **GAAAGAGTGGGGGAGAAAGG** |
| 3'ZEB2chr2:144993834-144994244 | **GAGTGGCCGAAAGAGATCAG** |
| 3'SMARCD3chr7:150603809-150605946 | **CCTTCACTCTGCAGGTAGGC** |
| 3'ID2chr2:8739658-8741083 | **ATAGTGGGATGCGAGTCCAG** |
| 3'CBX6chr22:37596886-37598202 | **ACCCAAACCCAAAACTTTCC** |
| 3'PDGFRAchr4:54788427-54789434 | **GCACCGCTCGCTATTACTTC** |
| 3'ESRP1chr8:95721437-95724616 | **CACAAACTTCTTGCCCCATT** |
| 3'OVOL1chr11:65311072-65312212 | **TCTTCCCTGAGAACGAGGTC** |
| 3'SYKchr9:92604055-92604413 | **AGCTCCAAGATCGACTTCCTC** |
| 3'CYP2R1chr11:14869178-14870388 | **ACGCGTGAATGAATTGATGA** |
| 3'CDH1chr16:67328383-67330049 | **GGTGTGGGAGTGCAATTTCT** |
| 3'FAM110Cchr2:34805-36603 | **ATGCTGCCAGAAAGCAGTTT** |
| 3'SGPP2chr2:222997519-222998089 | **GACTGCATTGAAAGCGTCTG** |
