# Supplementary figures and images for: Architecture of epigenetic reprogramming following Twist1-mediated epithelial-mesenchymal transition
Source: Genome Biol. 2013 Dec 24;14(12):R144. doi: 10.1186/gb-2013-14-12-r144 (PMC4053791; doi:10.1186/gb-2013-14-12-r144)

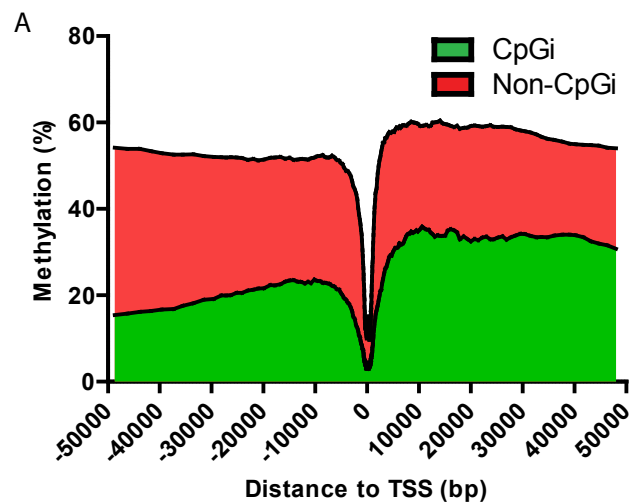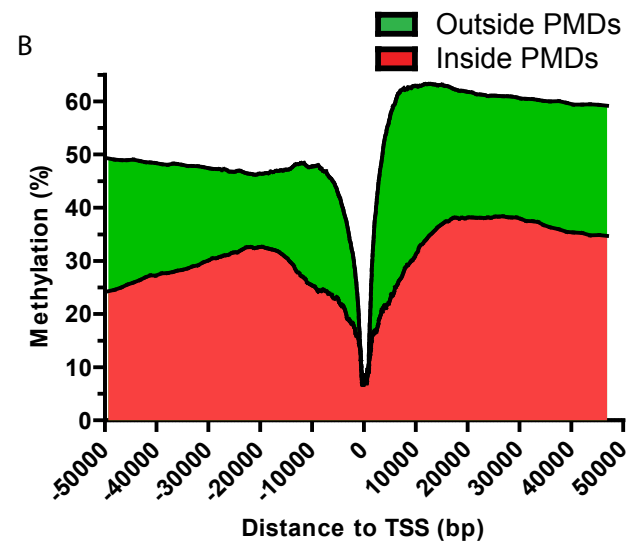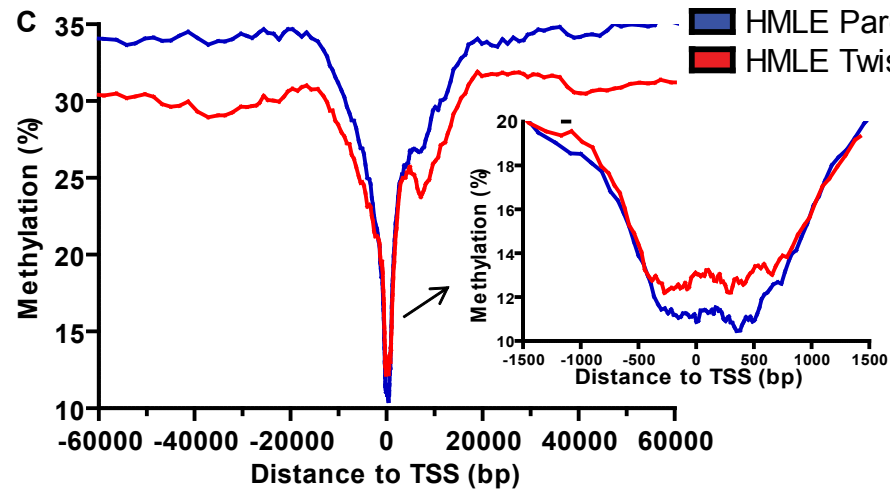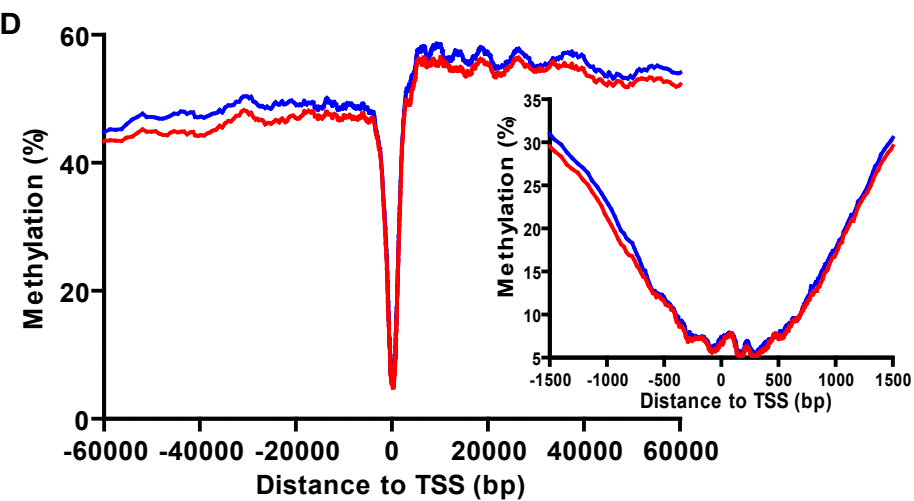

Supplement: Additional file 2: Figure S1 — Description of DREAM data. (A) DNA methylation level (y-axis) of CpG sites located in CGI (green) and non-CGI (red) according to the distance to the TSS (x-axis). Note the higher level of gene body methylation in comparison to the methylation levels of upstream regions. (B) DNA methylation level (y-axis) of CpG sites located in PMDs (red) and outside PMDs (green) according to the distance to TSS (x-axis). (C) Average DNA methylation levels (y-axis) of CpG sites located in PMDs of HMLE vector cells (blue) and HMLE Twist cells (red). x-axis represents distance of CpG sites to TSS. Note global DNA demethylation of PMDs in mesenchymal cells coupled with increased methylation at promoters. (D) Average DNA methylation levels (y-axis) of CpG sites located outside PMDs in HMLE vector cells (blue) and HMLE Twist cells (red). x-axis represents distance of CpG sites to TSS. Note the absence of global DNA demethylation as it is the case for DNA methylation within PMDs. [file gb-2013-14-12-r144-S2.pdf]

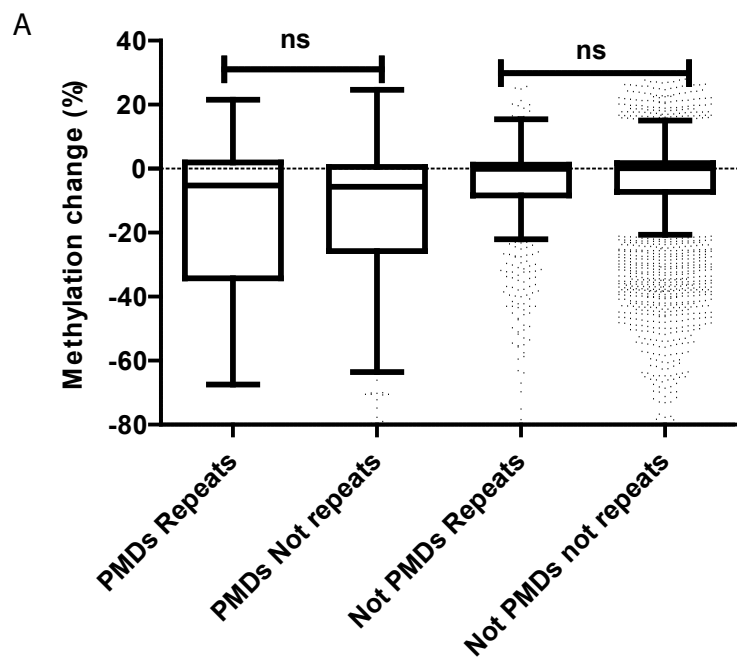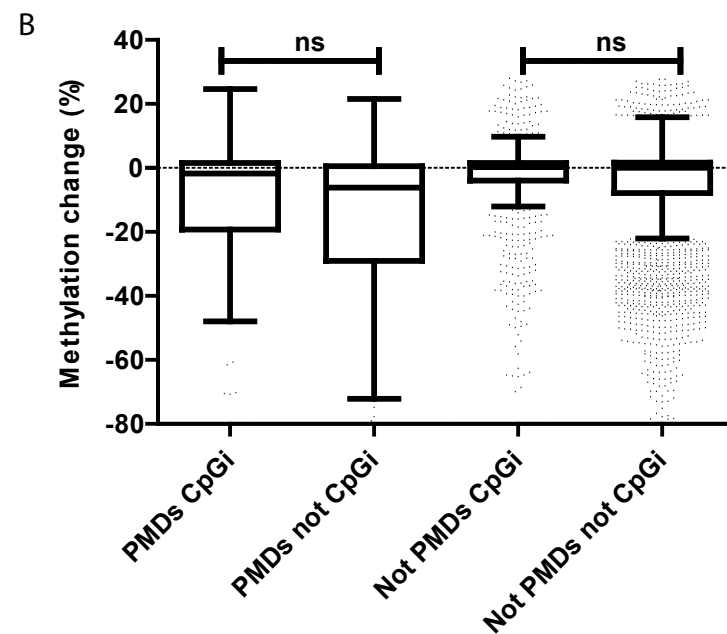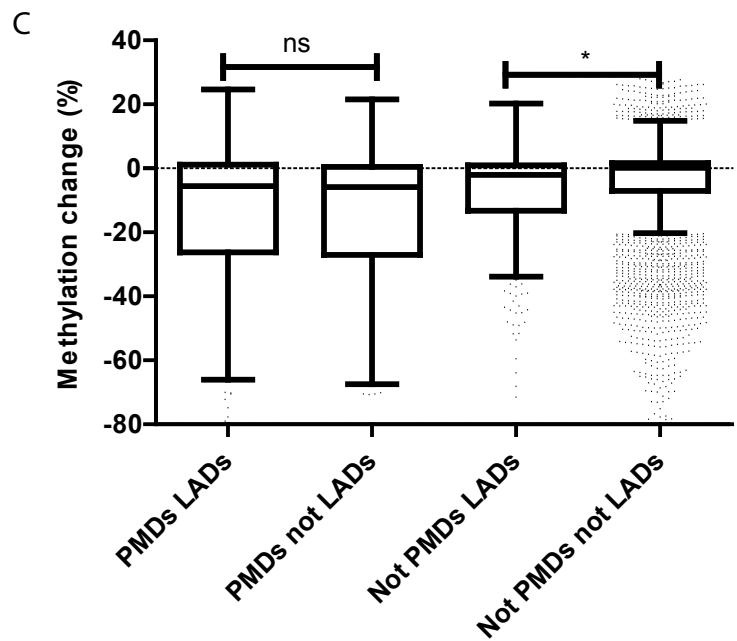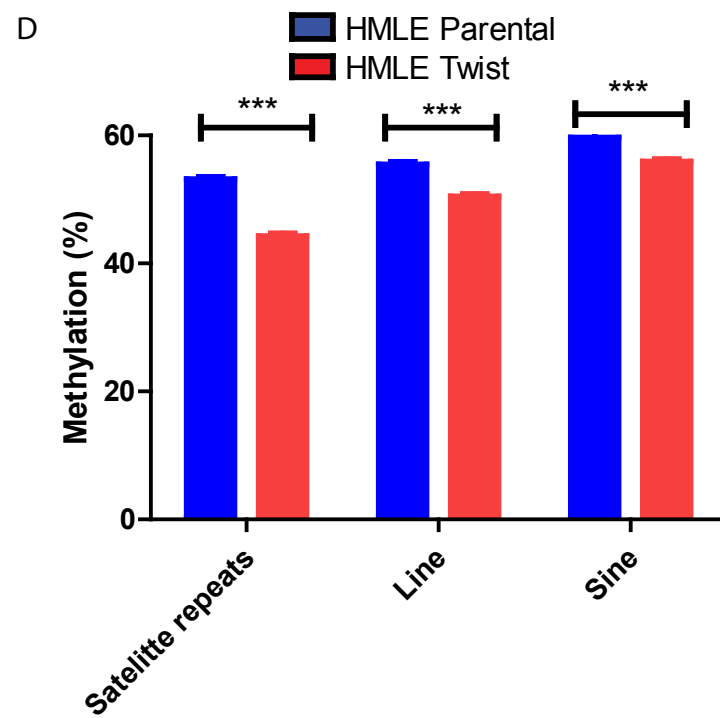

Supplement: Additional file 3: Figure S2 — Average DNA methylation levels in different genomic locations. (A) Average DNA methylation level of CpG sites located within/outside partially methylated domains (PMDs) and/or repetitive elements. ns stands for not significant. * P <0.05. (B) Average DNA methylation level of CpG sites located within/outside PMDs and/or CpG islands. (C) Average DNA methylation level of CpG sites located within/outside PMDs and/or lamina associated domains. (D) Average DNA methylation level of CpG sites located in repetitive elements of HMLE vector cells (blue) and in HMLE Twist cells cultured in monolayer. ***P <0.0001. [file gb-2013-14-12-r144-S3.pdf]

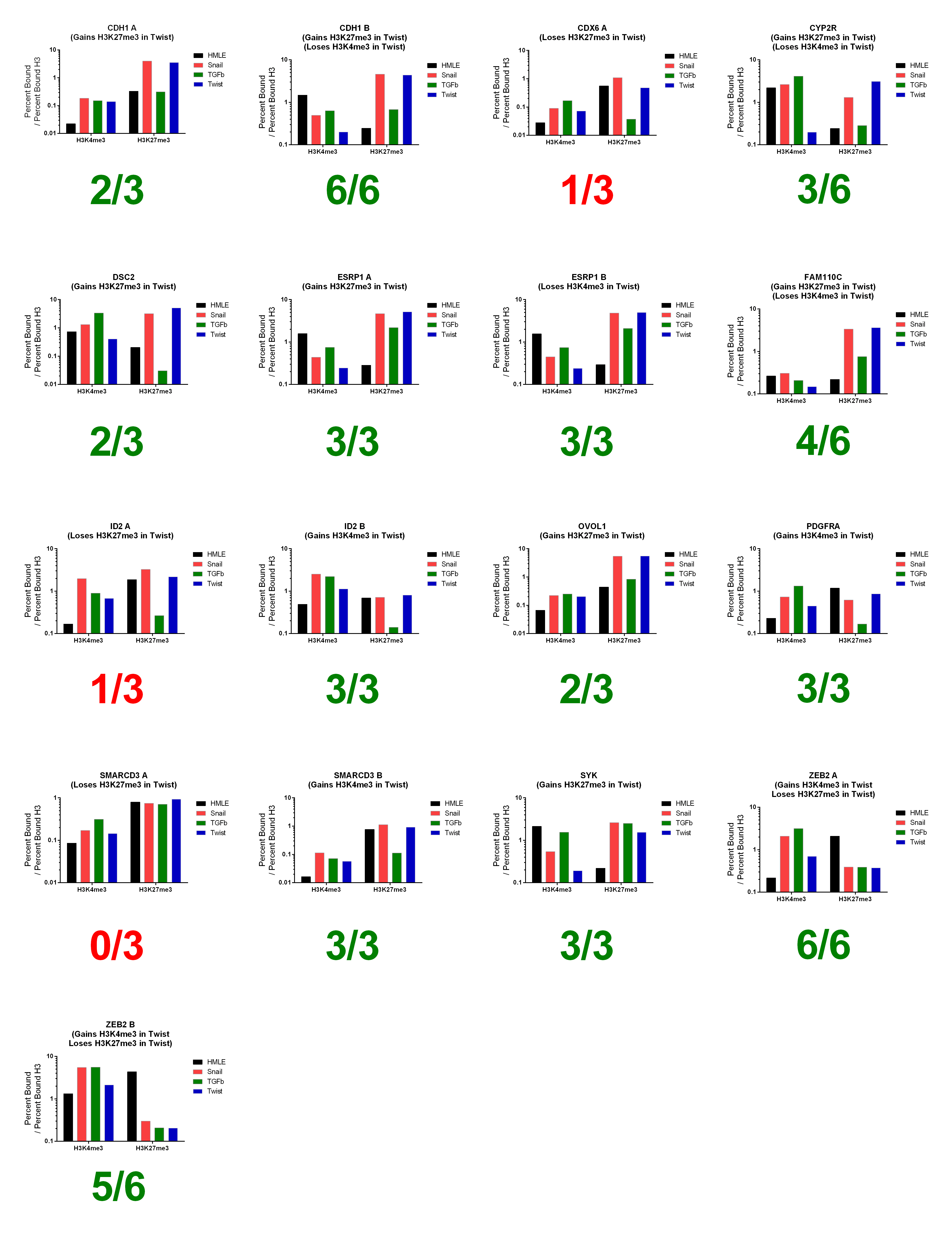

Supplement: Additional file 5: Figure S4 — ChIP-qPCR results for H3K4me3 and H3K27me3 in 17 loci in HMLE vector cells, and HMLE Twist-, Snail- and TGF-β1-mediated EMT. [file gb-2013-14-12-r144-S5.jpeg]

## Slide 1
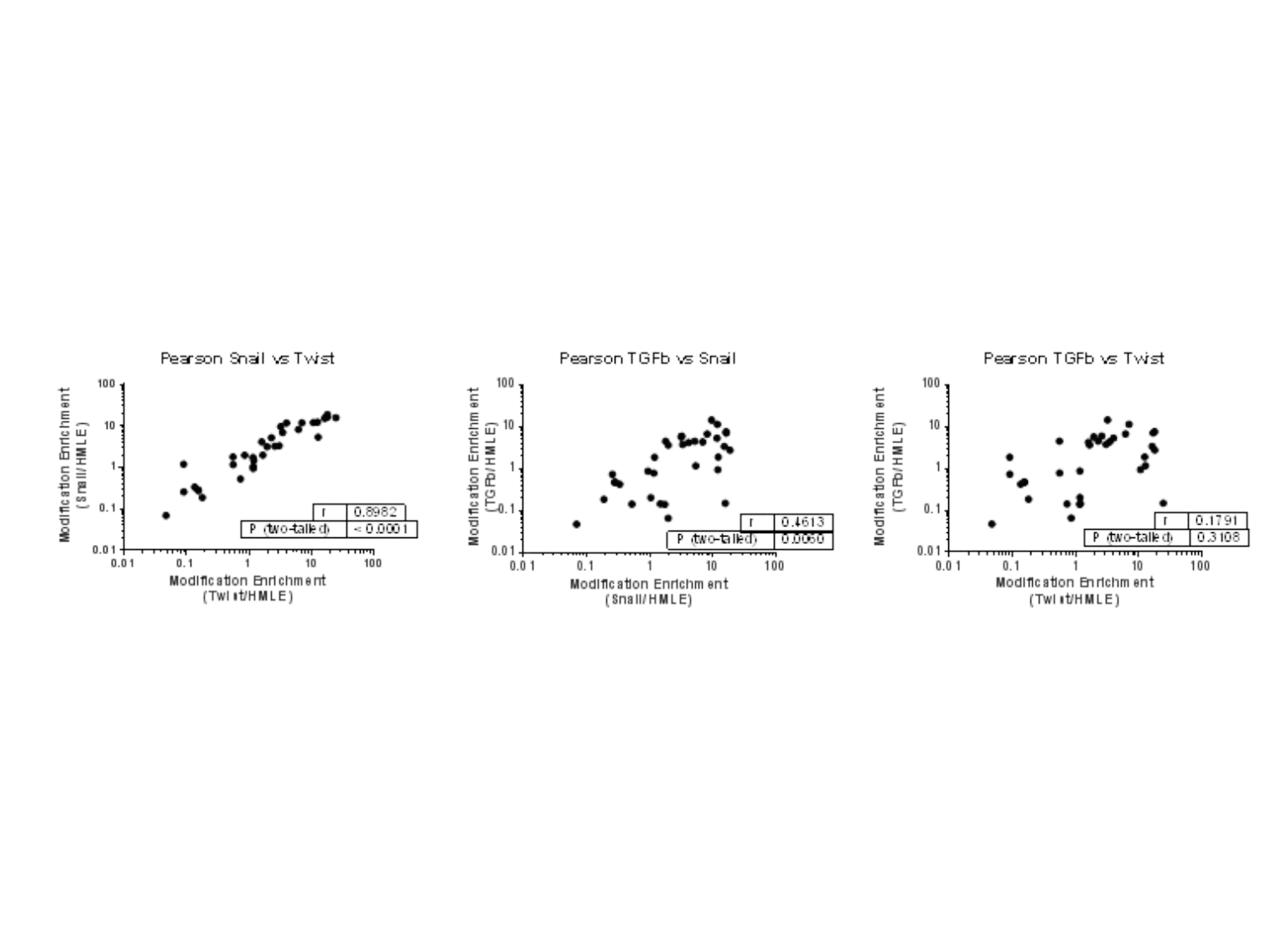

Supplement: Additional file 6: Figure S5 — Pearson correlation of ChIP-qPCR results between HMLE Twist, Snail and TGF-β1 cells. [file gb-2013-14-12-r144-S6.pptx]

## Slide 1
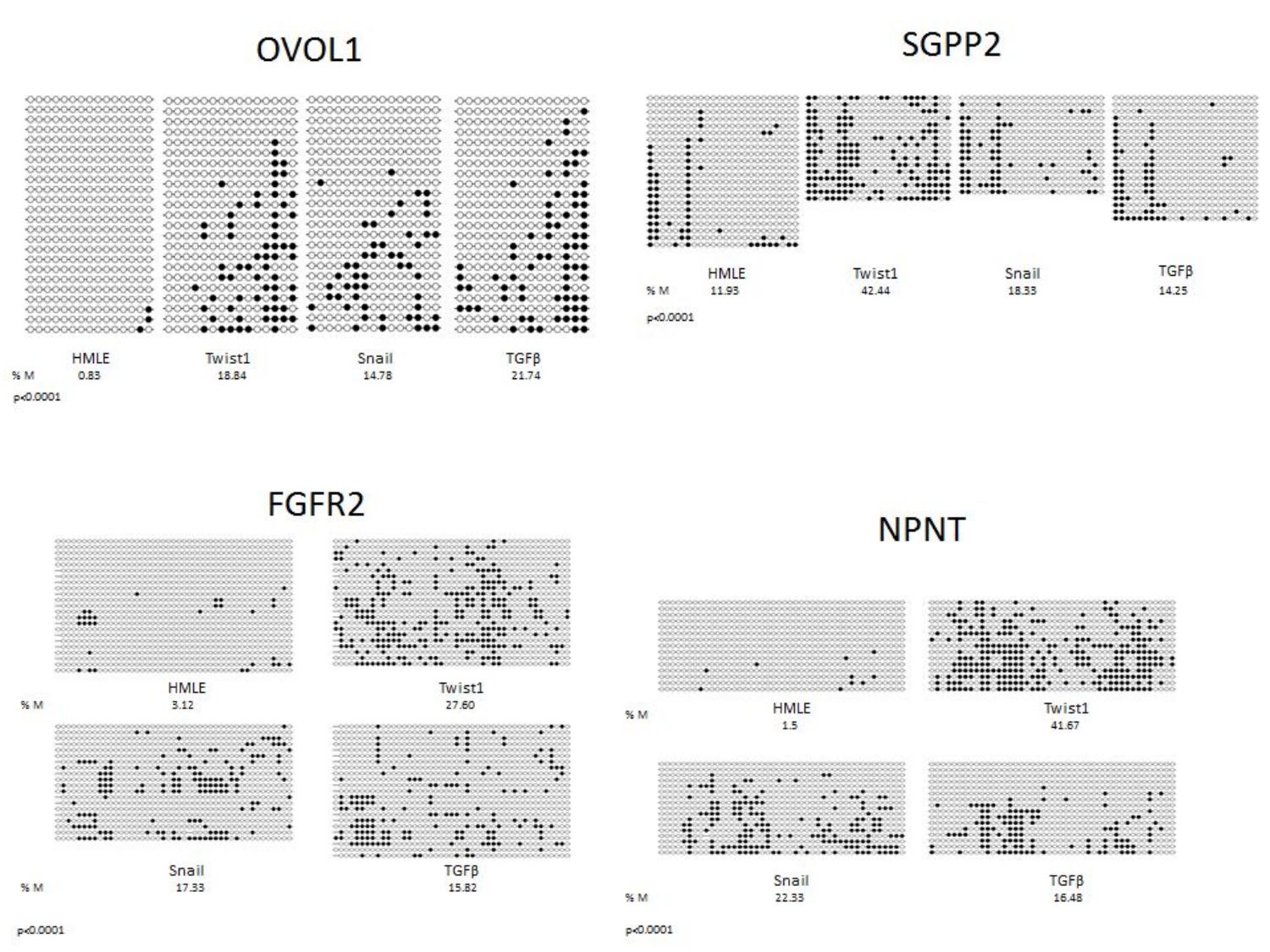

Supplement: Additional file 7: Figure S6 — DNA methylation changes using bisulfite sequencing in seven selected gene promoters following Twist-, Snail- and TGF-β1-mediated EMT. [file gb-2013-14-12-r144-S7.pptx]

## Slide 1
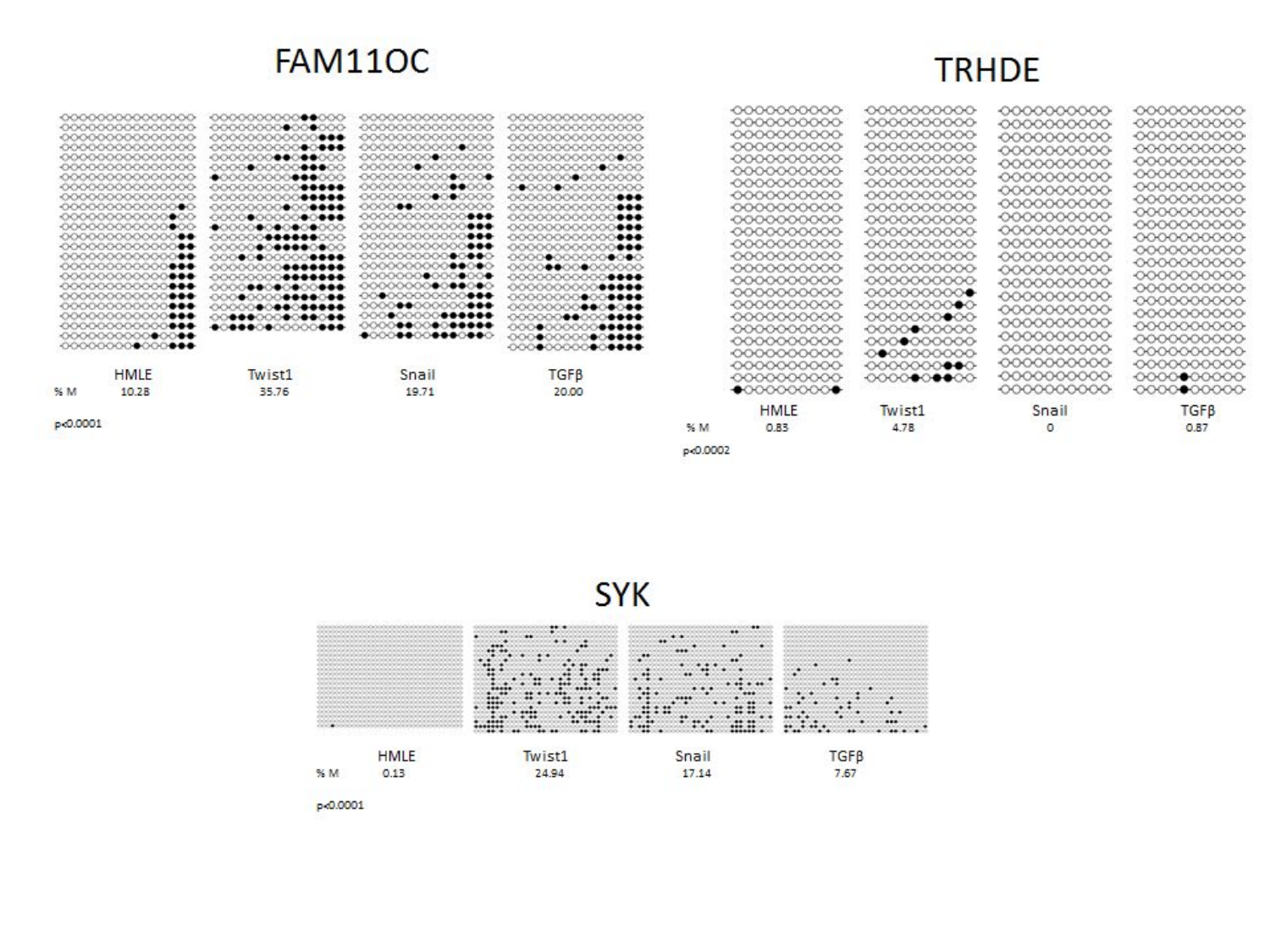

Supplement: Additional file 8: Figure S7 — DNA methylation changes using bisulfite sequencing in seven selected gene promoters following Twist-, Snail- and TGF-β1-mediated EMT. [file gb-2013-14-12-r144-S8.pptx]

## Slide 1
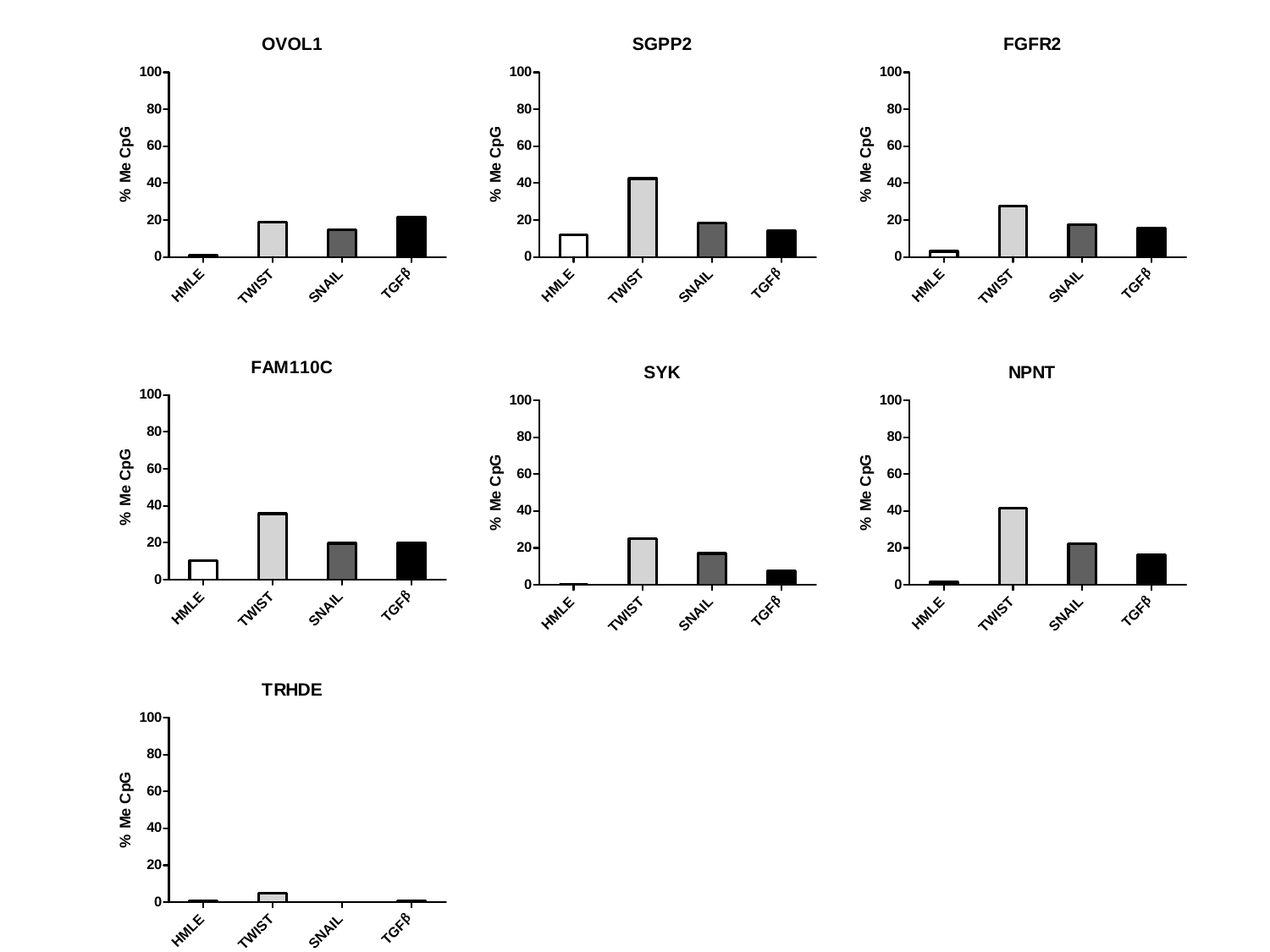

Supplement: Additional file 9: Figure S8 — DNA methylation changes using bisulfite sequencing in seven selected gene promoters following Twist-, Snail- and TGF-β1-mediated EMT. [file gb-2013-14-12-r144-S9.pptx]

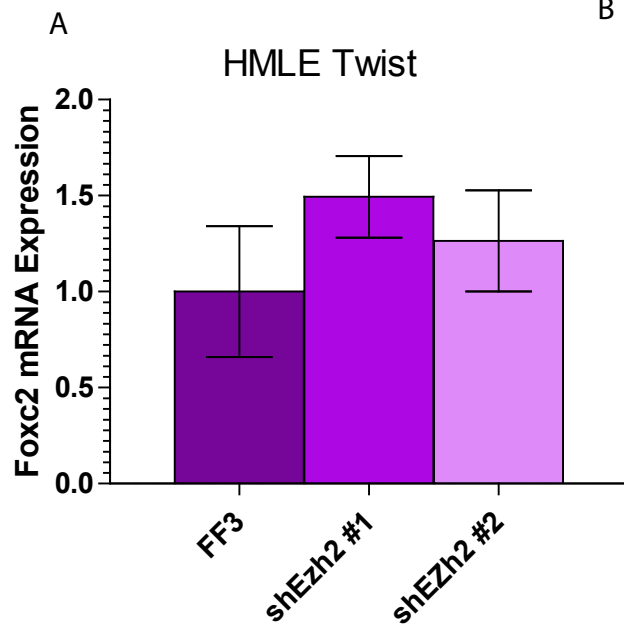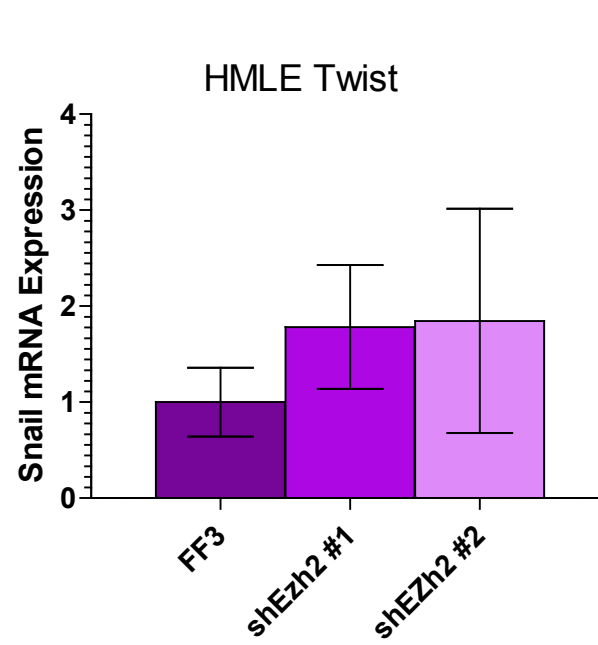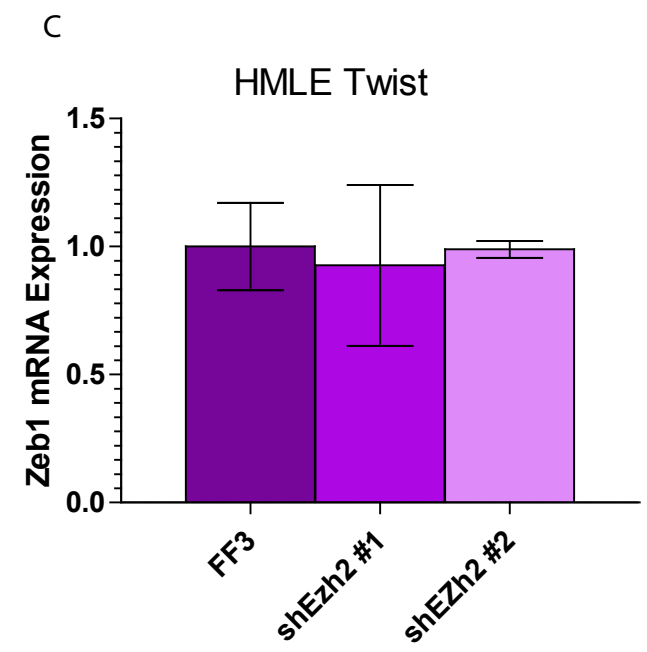

Supplement: Additional file 11: Figure S10 — Expression of EMT-related transcription factors after EZH2 knockdown. qRT-PCR performed for FOXC2(A), SNAIL(B) and ZEB1(C) using RNA extracted from control and shEZH2 cells showed that the expression level of those EMT-related transcription factors remain unchanged. [file gb-2013-14-12-r144-S11.pdf]

A

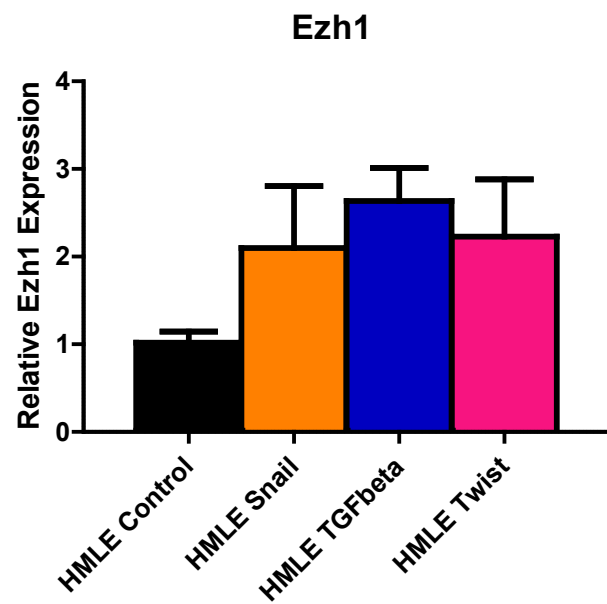

B

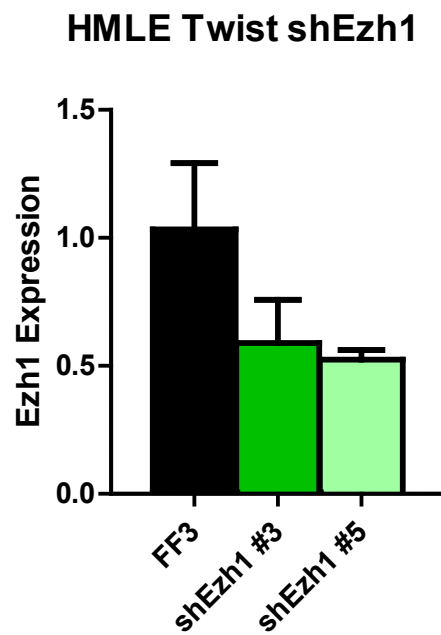

C

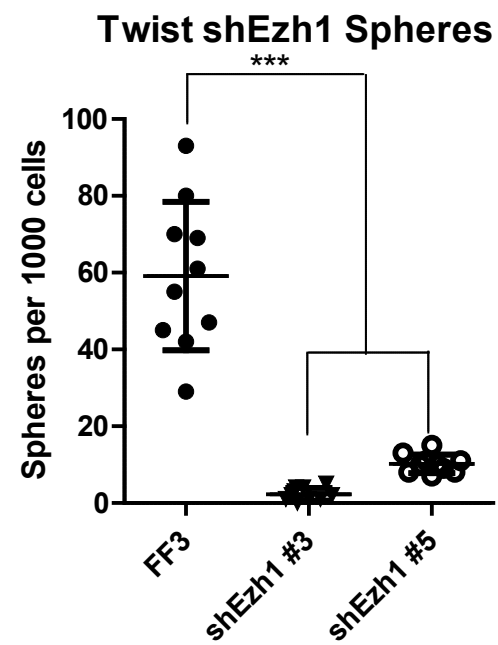

Supplement: Additional file 12: Figure S11 — EZH1 knockdown in HMLE Twist cells. (A) qRT-PCR expression of EZH1 in the indicated cell lines. (B) Knockdown of EZH1 mediated by two independent shRNAs. EZH1 mRNA expression was quantified by RT-PCR in the indicated cell lines. (C) Control and shEZH1 cells were grown in MS-promoting conditions and spheres greater in size than 50 μm were counted after 10 days. A Student’s t-test was performed. [file gb-2013-14-12-r144-S12.pdf]

Vimentin

DAPI

Merge

HMLE

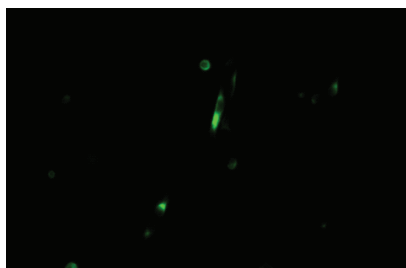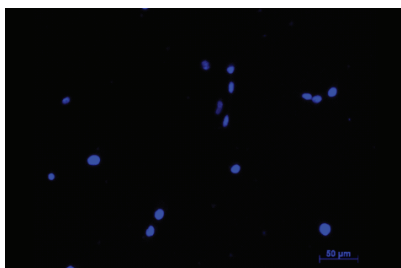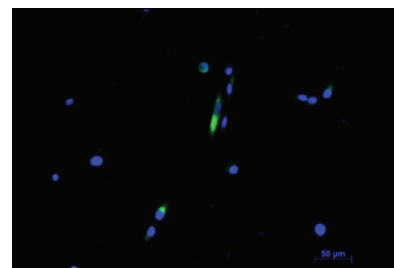

Twist

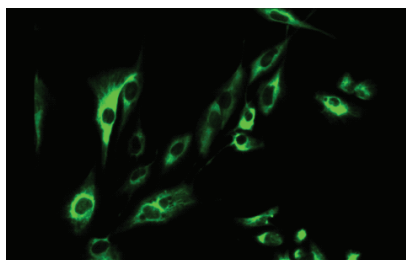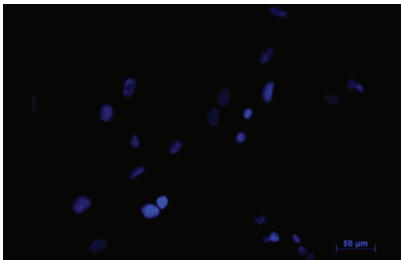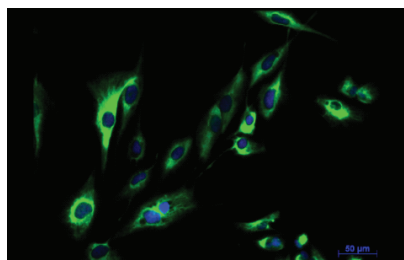

FOXC2

DAPI

Merge

HMLE

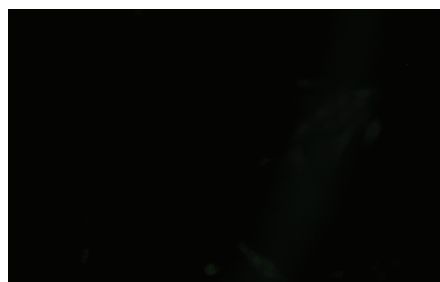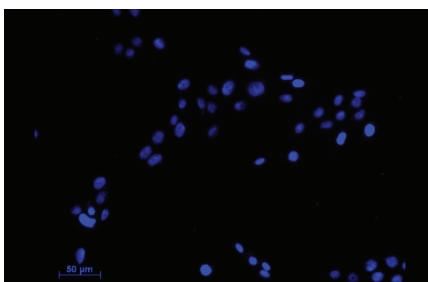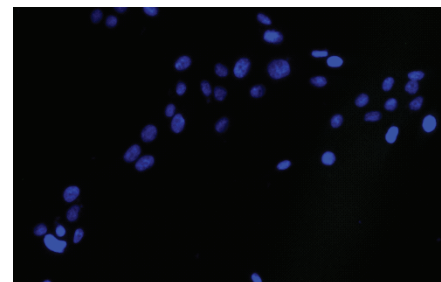

Twist

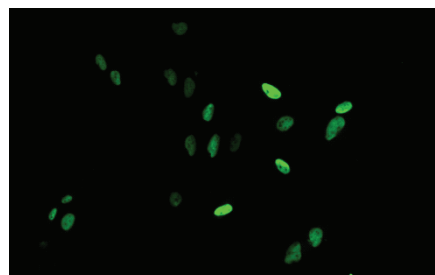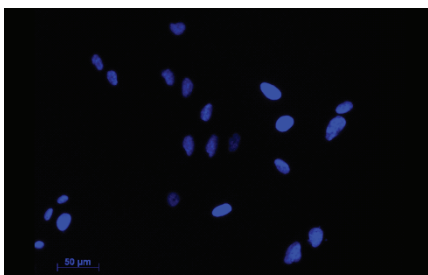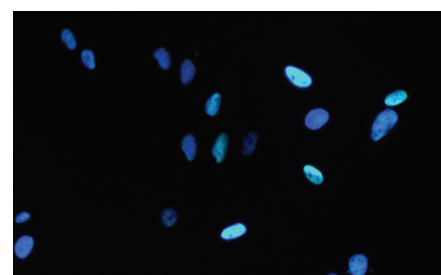

Supplement: Additional file 13: Figure S12 — Twist expression generated cells with consistent levels of vimentin and Foxc2 expression. HMLE vector and HMLE Twist cells were stained for vimentin (a) and for Foxc2 (b) along with a DAPI co-stain. [file gb-2013-14-12-r144-S13.pdf]

## Slide 1
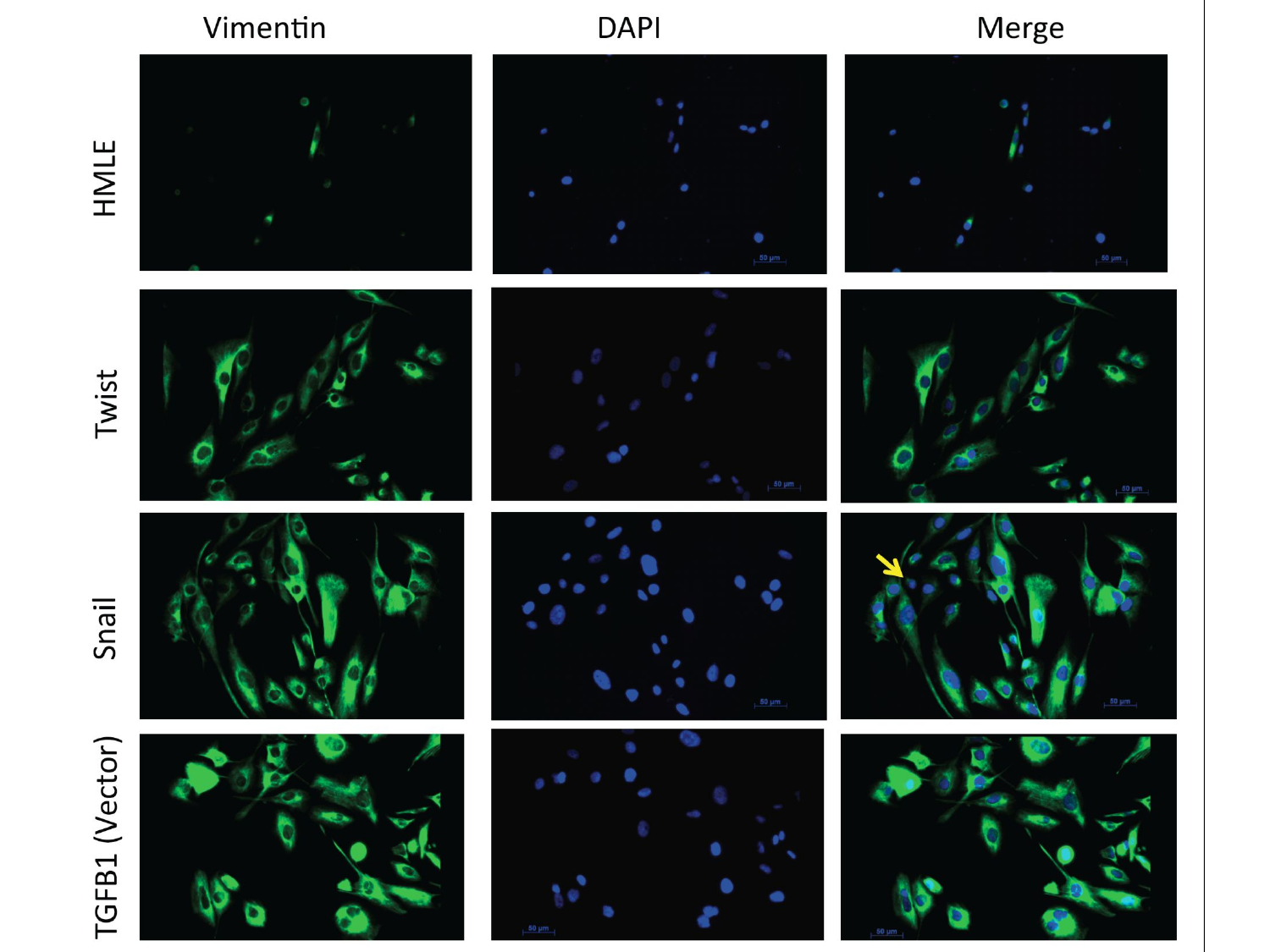

Supplement: Additional file 14: Figure S13 — Twist, Snail and TGF-β1 expression generated cells with consistently elevated levels of vimentin. HMLE vector, HMLE Snail, HMLE Twist and HMLE TGF-β1 cells were stained for vimentin along with a DAPI co-stain. The yellow arrow indicates a cell without appreciable expression of vimentin. [file gb-2013-14-12-r144-S14.pptx]
