# Supplementary material for: Architecture of epigenetic reprogramming following Twist1-mediated epithelial-mesenchymal transition
Source: Genome Biol. 2013 Dec 24;14(12):R144. doi: 10.1186/gb-2013-14-12-r144 (PMC4053791; doi:10.1186/gb-2013-14-12-r144)
Supplement: Additional file 4: Figure S3 — Histone switches of highly up-regulated and down-regulated genes in HMLE Twist cells as compared to HMLE vector cells. The majority of genes that become up-regulated in mesenchymal cells and were pre-marked by H3K27me3 in vector cells switched to H3K4me3 in HMLE Twist cells. Conversely, the majority of genes that become down-regulated in mesenchymal cells and were pre-marked by H3K4me3 in vector cells switched to H3K27me3. [file gb-2013-14-12-r144-S4.pdf]

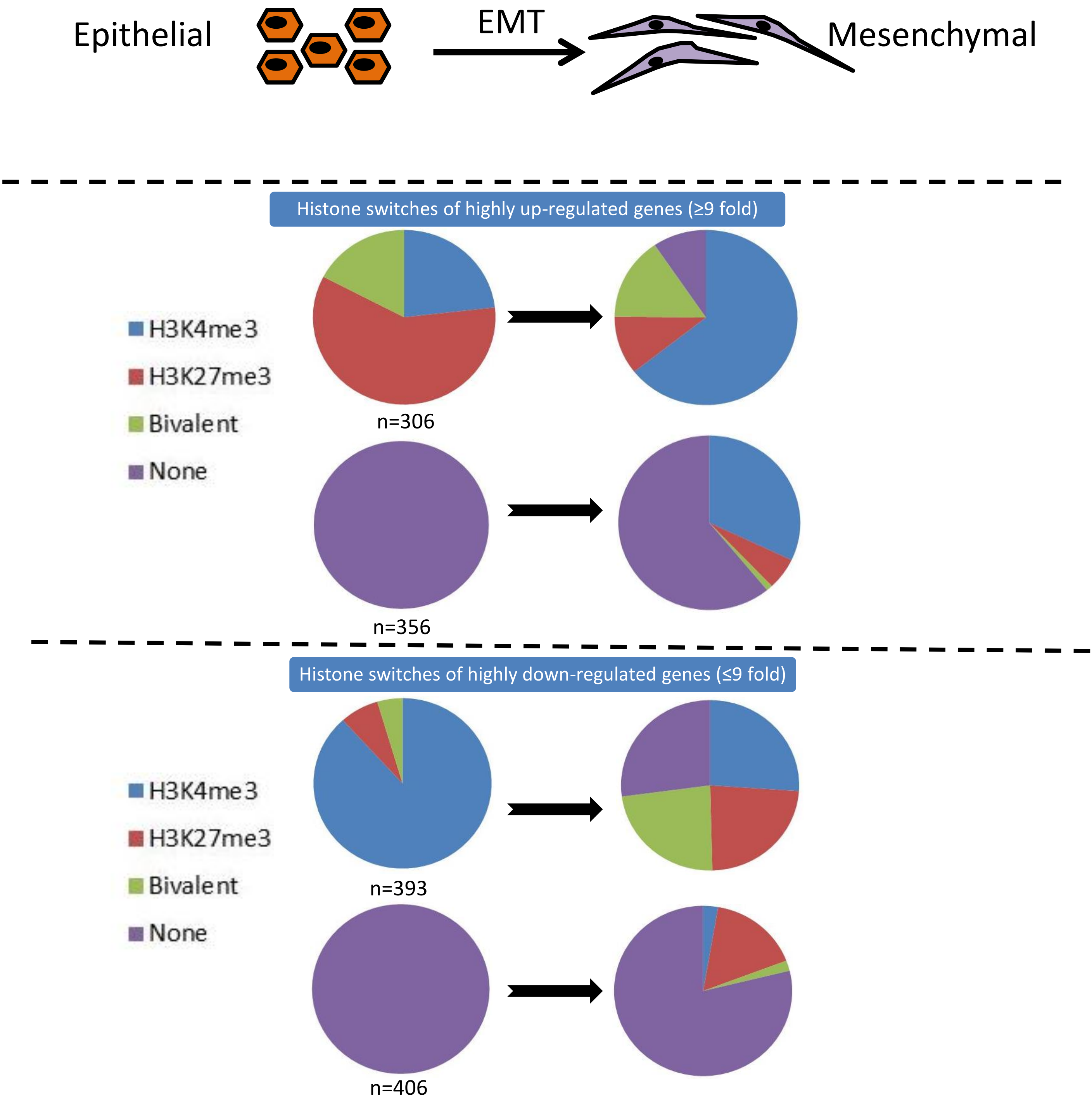
Epithelial
EMT
Mesenchymal
Histone switches of highly up-regulated genes (≥9 fold)
H3K4me3
H3K27me3
Bivalent
None
n=306
n=356
Histone switches of highly down-regulated genes (≤9 fold)
H3K4me3
H3K27me3
Bivalent
None
n=393
n=406
