# Supplementary material for: Architecture of epigenetic reprogramming following Twist1-mediated epithelial-mesenchymal transition
Source: Genome Biol. 2013 Dec 24;14(12):R144. doi: 10.1186/gb-2013-14-12-r144 (PMC4053791; doi:10.1186/gb-2013-14-12-r144)

A

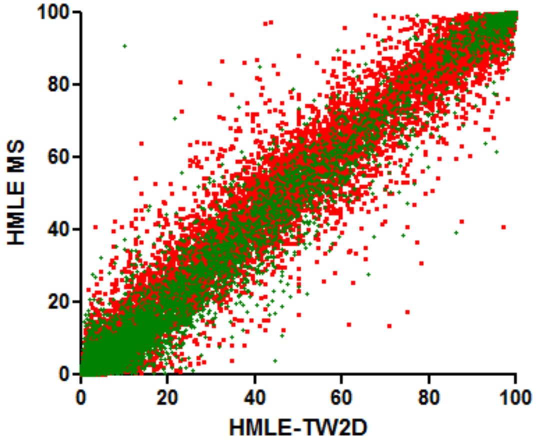


B

| MS \ 2D | H3K4me3 (n=17434) | H3K27me3 (n=2411) | Bivalent (n=1248) | None (n=12334) |
|---|---|---|---|---|
| H3K4me3 (n=14900) | 14338 | 6 | 349 | 207 |
| H3K27me3 (n=3607) | 186 | 1588 | 327 | 1506 |
| Bivalent (n=1626) | 592 | 317 | 509 | 208 |
| None (n=13294) | 2318 | 500 | 63 | 10413 |

C

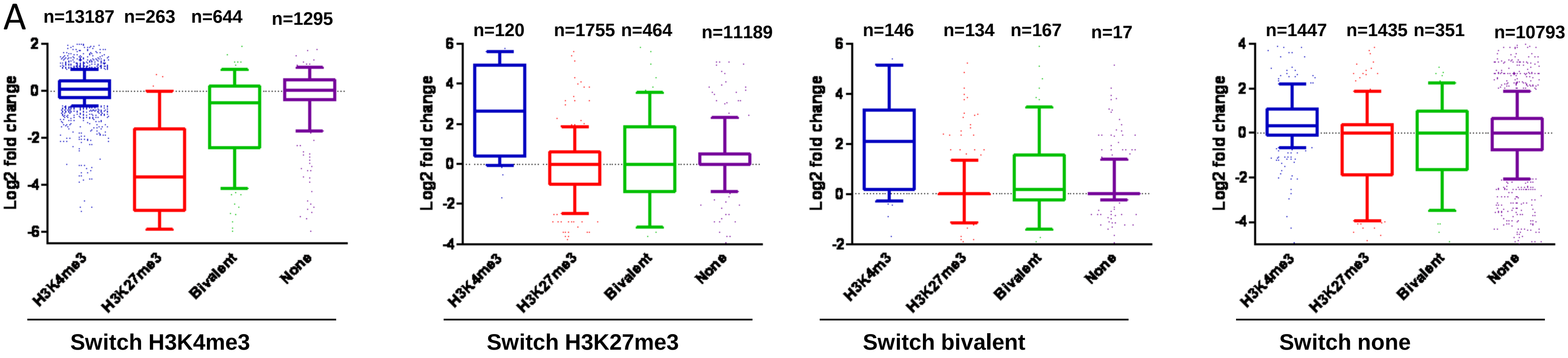

Supplement: Additional file 10: Figure S9 — DNA methylation and histone modifications in HMLE Twist cells cultured as spheres. (A) Box-plot of gene expression fold change (the bars represent 10% and 90% extreme) for genes switching in HMLE vector cells from H3K4me3, H3K27me3, bivalent or neither marks to other histone marks in HMLE Twist cells cultured as spheres. (B) Correlation of methylation level of CpG sites detected by DREAM in HMLE Twist cells cultured in a monolayer (2D) (x-axis) and as spheres (MS) (y-axis). Green, CpG sites located in CGI; red, CpG sites located outside CGI. (C) Switches of histone marks between HMLE Twist cells cultured in a monolayer and as spheres. [file gb-2013-14-12-r144-S10.pdf]
